# Modulation of Heat Shock Transcription Factor 1 as a Therapeutic Target for Small Molecule Intervention in Neurodegenerative Disease

**DOI:** 10.1371/journal.pbio.1000291

**Published:** 2010-01-19

**Authors:** Daniel W. Neef, Michelle L. Turski, Dennis J. Thiele

**Affiliations:** Department of Pharmacology and Cancer Biology, Duke University School of Medicine, Durham, North Carolina, United States of America; University of Wisconsin, United States of America

## Abstract

Neurodegenerative diseases such as Huntington disease are devastating disorders with no therapeutic approaches to ameliorate the underlying protein misfolding defect inherent to poly-glutamine (polyQ) proteins. Given the mounting evidence that elevated levels of protein chaperones suppress polyQ protein misfolding, the master regulator of protein chaperone gene transcription, HSF1, is an attractive target for small molecule intervention. We describe a humanized yeast-based high-throughput screen to identify small molecule activators of human HSF1. This screen is insensitive to previously characterized activators of the heat shock response that have undesirable proteotoxic activity or that inhibit Hsp90, the central chaperone for cellular signaling and proliferation. A molecule identified in this screen, HSF1A, is structurally distinct from other characterized small molecule human HSF1 activators, activates HSF1 in mammalian and fly cells, elevates protein chaperone expression, ameliorates protein misfolding and cell death in polyQ-expressing neuronal precursor cells and protects against cytotoxicity in a fly model of polyQ-mediated neurodegeneration. In addition, we show that HSF1A interacts with components of the TRiC/CCT complex, suggesting a potentially novel regulatory role for this complex in modulating HSF1 activity. These studies describe a novel approach for the identification of new classes of pharmacological interventions for protein misfolding that underlies devastating neurodegenerative disease.

## Abstract 2

A yeast-based small molecule screen identifies a novel activator of human HSF1 and protein chaperone expression and which appears to alleviate the toxicity of protein misfolding diseases.

## Author Summary

The misfolding of proteins into a toxic state contributes to a variety of neurodegenerative diseases such as Huntington, Alzheimer, and Parkinson disease. Although no known cure exists for these afflictions, many studies have shown that increasing the levels of protein chaperones, proteins that assist in the correct folding of other proteins, can suppress the neurotoxicity of the misfolded proteins. As such, increasing the cellular concentration of protein chaperones might serve as a powerful therapeutic approach in treating protein misfolding diseases. Because the levels of protein chaperones in the cell are primarily controlled by the heat shock transcription factor 1 [HSF1], we have designed and implemented a pharmacological screen to identify small molecules that can promote human HSF1 activation and increase the expression of protein chaperones. Through these studies, we have identified HSF1A, a molecule capable of activating human HSF1, increasing the levels of protein chaperones and alleviating the toxicity of misfolded proteins in both cell culture as well as fruit fly models of neurodegenerative disease.

## Introduction

Neuronal tissues are exquisitely sensitive to defective protein folding, and the accumulation of misfolded proteins is proteotoxic due to dominant effects of insolubility, inappropriate intermolecular interactions, and long half-lives. Protein misfolding is associated with neurodegenerative diseases that include Parkinson disease, amyotropic lateral sclerosis (ALS), transmissible spongiform encephalopathies (prion diseases), and other devastating diseases [1]. Hereditary protein conformational disorders are characterized by coding region trinucleotide expansions resulting in the insertion of poly-glutamine (polyQ) tracts that adopt β-sheet structures and that are prone to incorrect folding and aggregation [2]. To date, nine hereditary gain-of-function disorders including Huntington disease, dentatorubral-pallidoluysian atrophy, spinobulbar muscular atrophy, as well as six forms of spinocerebellar ataxia have been linked to polyQ expansions [2]. Although studies have suggested that amyloid formation observed in these states is intrinsic to the disease pathology, recent investigations suggest that the soluble oligomeric precursors of the large aggregates are the neurotoxic form [3]. Although there is no known cure for these devastating diseases, the ability to stabilize misfolded proteins into their native conformation would likely prevent the neuronal proteotoxicity that is observed in Huntington disease and other protein conformational disorders.

A variety of individual protein chaperones and cochaperone complexes function to fold, process, and degrade proteins, thereby playing a central role in cellular protein homeostasis [4]. Experiments in cell and animal models of neurodegenerative disease demonstrate that increased levels of individual protein chaperones such as Hsp70, Hsp40, or Hsp27 can significantly suppress protein aggregation, increase protein solubility and turnover, and ameliorate neuronal loss [5]–[16]. Additional studies suggest that simultaneous increases in Hsp70 and Hsp40 can synergize the suppression of polyQ-mediated neuronal degeneration [8],[9],[13]. Because most metazoan chaperones stabilize, but do not disaggregate misfolded proteins, these results are consistent with the oligomeric precursors of amyloid fibrils being toxic to neurons, rather than the aggregates themselves [3],[14],[15],[17].

In eukaryotic cells, multiple genes encoding protein chaperones are coordinately transcriptionally activated in response to proteotoxic conditions, such as acute increases in temperature, by the heat shock transcription factor 1 (HSF1) protein and *cis*-acting promoter sequences called heat shock elements (HSEs) [18]–. As such, protein chaperones are often referred to as heat shock proteins, or Hsps. Although the mechanisms for HSF1 activation are incompletely understood, a multistep process occurs in response to stress that involves the interconversion of an inactive cytoplasmic monomer to a homotrimer, nuclear accumulation, DNA binding to HSEs, hyperphosphorylation, and target gene *trans*-activation [21]. Studies suggest that under low proteotoxic stress conditions, HSF1 is largely an inactive cytoplasmic monomer that is bound to Hsp90, Hsp70, and other proteins in a repressive complex [22],[23]. It is thought that under stress conditions, HSF1 dissociates from this complex, allowing homomultimerization and activation. Although the association of HSF1 with protein chaperones is one mechanism for its regulation, HSF1 is also thought to engage in intramolecular coiled-coil interactions that maintain the monomeric state and point mutations in these coiled-coil domains (leucine zippers) cause constitutive multimerization when expressed in mammalian cells [24]. Studies also suggest that HSF1 has intrinsic stress-sensing capacity, as both *Drosophila* and mammalian HSF1 can be converted from a monomer to a homotrimer in vitro in response to thermal or oxidative stress [25]–[27].

Previous reports demonstrate that the conversion of HSF1 to the high-affinity DNA binding homotrimer is not robust in neuronal cells [28]. Although the precise mechanisms underlying this defect in HSF1 activation are not clear, this could, in part, explain the selective sensitivity of neuronal cells in neurodegenerative diseases in which misfolded proteins are expressed in all tissues [28]. A recent report demonstrated that a cellular model and a mouse model of Huntington disease expressing a constitutively active form of human HSF1 exhibited reduced polyglutamine protein aggregation [5]. Furthermore, the expression of activated HSF1 in nonneuronal tissues prolonged the lifespan of this mouse model of Huntington disease. Yeast cells harboring an HSF molecule partially defective in *trans*-activation were shown to promote yeast prion formation, implying a potential role for HSF target gene products in the prevention of prion generation or propagation [29]. Moreover, *hsf1^−/−^* mice inoculated with Rocky Mountain Laboratory prions exhibited a shorter lifespan as compared to wild-type mice [30]. Taken together, many studies support the value of individual protein chaperone expression, the synergistic expression of multiple protein chaperones, or the expression of an activated form of HSF1, in cellular, worm, fly, and mouse models of protein misfolding. Given the potential therapeutic role of elevated protein chaperone levels in diseases of protein conformation and the coordinated expression of Hsps via the action of human HSF1, small molecule activation of human HSF1 is likely to be a promising avenue for therapeutic intervention in neurodegenerative disease.

Previous screens utilizing an HSF1-dependent reporter gene as a readout for HSF1 activation in mammalian cells have identified activators of HSF1 [31]. However, these screens often result in the identification of compounds that promote HSF1 activation through the proteotoxic accumulation of unfolded proteins or through the inhibition of Hsp90, a central chaperone involved in cell growth, signaling, and proliferation [32]–[36]. As such, novel approaches are needed to identify novel compounds that promote HSF1 activation without the inhibition of Hsp90 or promoting the accumulation of unfolded proteins. Here, we describe a novel high-throughput screen to identify small molecule activators of human HSF1 using the budding yeast *Saccharomyces cerevisiae*. This screen, insensitive to established proteotoxic agents and Hsp90 inhibitors, identifies novel small molecules that activate HSF1 in the amelioration of neurodegenerative phenotypes in metazoan models of polyQ-based disease.

## Results

### A Humanized Yeast Screen for Small Molecule Activators of HSF1

**Figure 1:**
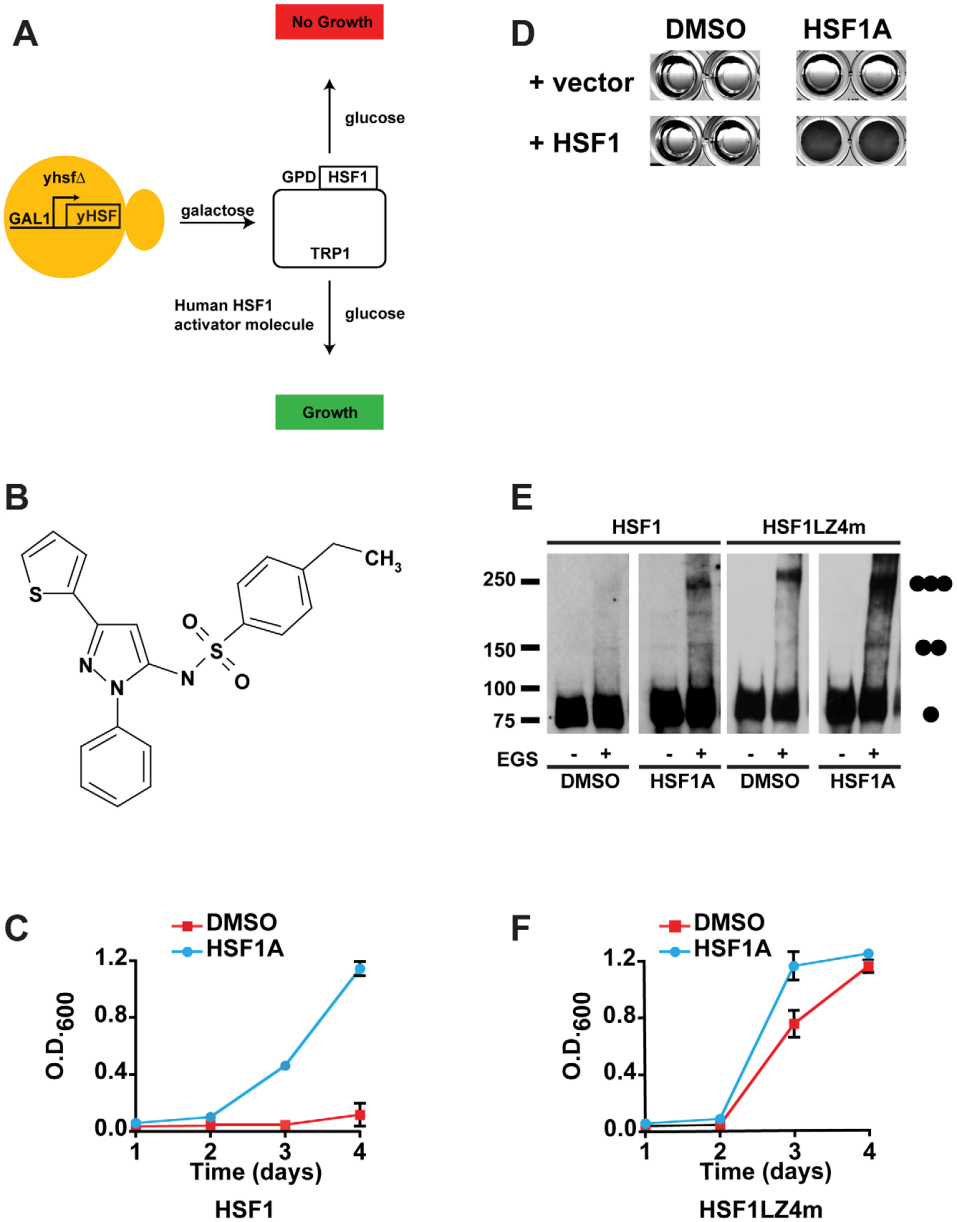
HSF1A activates human HSF1 function in yeast. (A) Strategy used to identify chemical activators of human HSF1 in yeast. Yeast cells expressing the essential yeast HSF under control of the glucose-repressible *GAL1* promoter are dependent on galactose for growth. Upon shifting cells to glucose-containing medium, the cells are dependent on activation of human HSF1 for growth. (B) Structure of HSF1A. (C) Yeast cells expressing wild-type human HSF1 were supplemented with 10 µM HSF1A or DMSO and grown in 96-well plates for 4 d. Growth was monitored by measuring OD_600_. (D) HSF1-dependence for HSF1A-mediated cell growth. Strain DNY75 expressing either human HSF1 (+HSF1) or an empty vector (+vector) were seeded into microtiter wells and incubated in the presence of HSF1A or DMSO solvent for 4 d and then photographed. Note that only cells expressing human HSF1 grow in response to HSF1A. (E) Yeast strain DNY75 was grown in the presence of DMSO or 20 µM HSF1A for 18 h, and HSF1 multimerization was evaluated by EGS cross-linking, SDS-PAGE, and immunoblotting. The positions of molecular weight markers are indicated on the left, and circles indicating the expected migration of HSF1 monomers, dimers, and trimers are on the right. (F) Yeast cells expressing HSF1LZ4m were supplemented with 10 µM HSF1A or DMSO and grown in 96-well plates for 4 d. Growth was monitored by measuring OD_600_.

We previously demonstrated that expression of human HSF1 is unable to suppress the viability defect of *S. cerevisiae* cells lacking endogenous HSF [37]. Moreover, human HSF1 was not activated in yeast in response to proteotoxic conditions such as heat shock that potently activate protein chaperone expression in mammalian cells. Biochemical analysis and functional complementation by an HSF1 mutant that is constitutively trimerized in human cells (HSF1LZ4m) indicated that the functional defect lies in the inability of human HSF1 to homotrimerize in yeast cells. As HSF1 multimerization is an early and essential step in the HSF1 activation program in human cells, we utilized the inability of human HSF1 to multimerize in yeast to screen for small molecule activators of HSF1 that would support yeast cell growth (Figure 1A).

For this humanized yeast screen, an *S. cerevisiae* strain was created harboring a deletion of the chromosomal yeast HSF locus (*yhsf*Δ) and which expresses a wild-type episomal copy of yeast HSF from the galactose-inducible, glucose-repressible *GAL1* promoter. Moreover, this strain expresses the wild-type human HSF1 protein from the plasmid-borne constitutive GPD promoter. To maximize small molecule uptake and retention, this strain was further modified by deleting the genes encoding the drug efflux pumps, Pdr5 and Snq2, as well as the gene encoding Erg6, an enzyme involved in ergosterol biosynthesis, which increases the permeability of small molecules through the plasma membrane, to create strain DNY75 [38],[39].

Strain DNY75 was grown to mid-log phase in galactose-containing medium to drive expression of yeast HSF and then shifted to dextrose-containing medium to extinguish expression of yeast HSF, rendering growth entirely dependent on the activation of human HSF1. Cells were seeded at low density to 96-well microtiter dishes and incubated with either DMSO solvent or an aliquot of a small molecule library. A nonbiased chemical library of over 10,000 compounds [40], built on approximately 70 scaffold structures, was screened at a final concentration of 10 µM for their ability to activate HSF1-dependent growth over a 4-d period at 30°C, as assayed spectrophotometrically. Positive hits from the primary screen were retested in two subsequent screens at the same concentration. Two benzyl pyrazole-based molecules, which we have named HSF1A and HSF1C (Figure 1B and Figure S1A), were potent activators of human HSF1-depenent yeast growth (Figure S1B). Because HSF1A was among the most potent activators of human HSF1-dependent yeast growth identified in our assay, it was chosen for further investigation.

HSF1A was synthesized de novo to independently confirm its ability to activate human HSF1-dependent yeast cell growth. As shown in Figure 1C, HSF1A, but not DMSO solvent, stimulated HSF1-dependent growth for strain DNY75. HSF1A was unable to support growth of a *yhsf*Δ yeast strain in the absence of human HSF1, confirming that HSF1A functions in an HSF1-dependent manner to support yeast cell growth (Figure 1D). Because the inability of HSF1 to complement the viability defect of *yhsf*Δ cells is due to defective HSF1 multimerization in yeast, cross-linking experiments were conducted to ascertain whether HSF1A promotes increased HSF1 multimerization in yeast. Although HSF1 multimers were not detected in lysates from cells treated with DMSO, HSF1 homotrimerization is detected when yeast cells were treated with HSF1A (Figure 1E). The constitutive trimerization of the HSF1LZ4m protein [37] was also further increased after HSF1A exposure, consistent with the observation that HSF1A also enhances LZ4m-dependent yeast growth (Figure 1F).

### HSF1A Coordinately Activates Protein Chaperone Expression in Mammalian Cells

**Figure 2:**
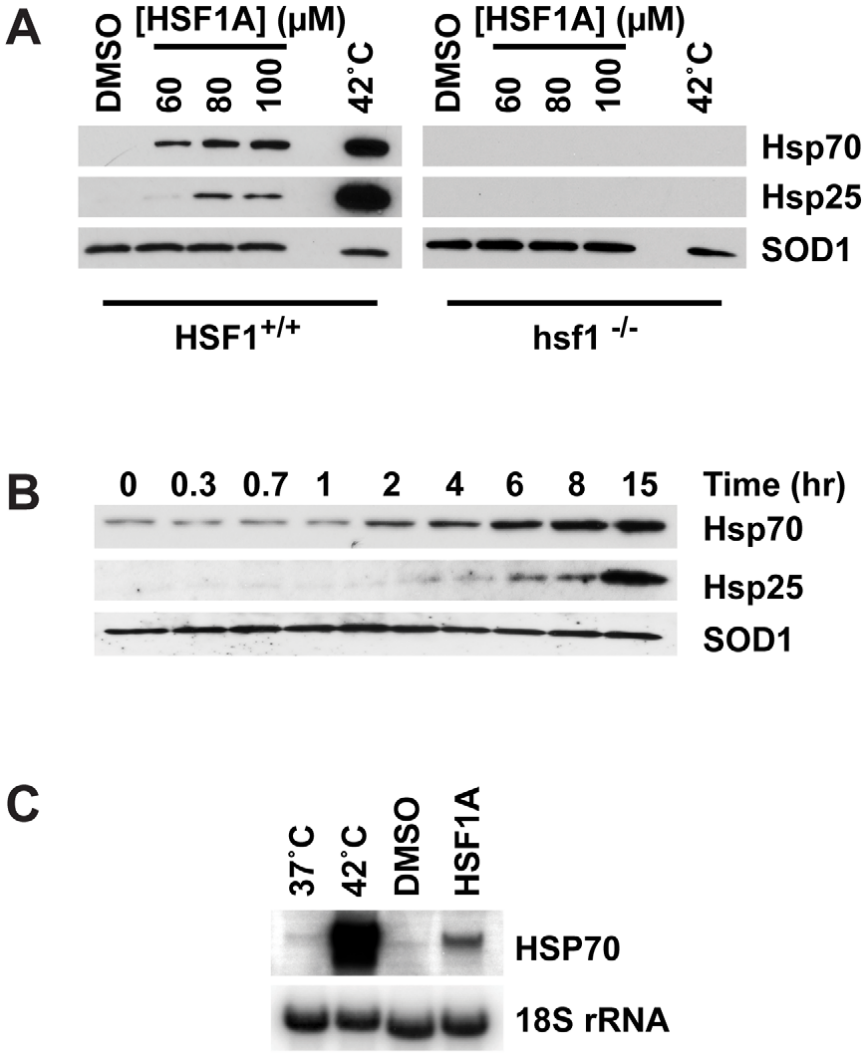
HSF1A activates Hsp70 expression in mammalian cells. (A) HSF1^+/+^ and *hsf1^−/−^* MEFs were treated with DMSO solvent or increasing concentrations of HSF1A for 15 h or heat shocked for 2 h at 42°C followed by a 15-h recovery. Total protein was analyzed for Hsp70 and Hsp25 expression by immunoblotting. SOD1 serves as a loading control. (B) HSF1^+/+^ MEF cells were treated with 80 µM HSF1A over time and Hsp70 and Hsp25 levels analyzed by immunoblotting. (C) HSF1^+/+^ MEF cells were treated with 80 µM HSF1A for 6 h or heat shocked at 42°C for 2 h, and total RNA was analyzed by RNA blotting. 18S rRNA serves as a loading control.

HSF1A stimulates human HSF1 multimerization in yeast and activates human HSF1-dependent yeast cell growth. To test whether HSF1A is able to activate HSF1 in mammalian cells, wild-type and *hsf1^−/−^* mouse embryonic fibroblasts (MEFs) [41] were incubated in the presence of DMSO solvent or increasing concentrations of HSF1A and the expression of two protein chaperones, Hsp70 and Hsp25, assessed by immunoblotting. Although DMSO did not enhance protein chaperone expression, treatment of wild-type MEFs with HSF1A resulted in the activation of Hsp70 and Hsp25 in a dose-dependent manner (Figure 2A). HSF1A-dependent activation of Hsp70 and Hsp25 was not detected in *hsf1^−/−^* cells, demonstrating that HSF1A functions in the activation of mammalian protein chaperones through HSF1. As previously demonstrated, wild-type MEFs exhibited robust expression of Hsp70 and Hsp25 in response to an acute heat shock that was absent in *hsf1^−/−^* MEFs. Interestingly, although HSF1C was also able to promote Hsp70 activation in mammalian cells, it did so at lower potency than HSF1A, mimicking the human HSF1 activation potential of both molecules in yeast (Figure S1C).

HSF1A-dependent activation of Hsp70 expression was also observed in HeLa cells, confirming its ability to activate HSF1 in human cell lines (Figure S2). Expression of Hsp70 and Hsp25 was detected in wild-type MEFs by approximately 2 h and was sustained for at least 15 h in the chronic presence of HSF1A (Figure 2B). Moreover, HSF1A treatment elevated *Hsp70* mRNA levels, consistent with HSF1A activating *Hsp70* gene transcription through HSF1 (Figure 2C). Although higher concentrations of HSF1A were used in these experiments, ELISA experiments demonstrated dose-dependent activation of Hsp70 at lower concentrations (Figure S3).

### HSF1A Promotes HSF1 Nuclear Localization and Hyperphosphorylation

**Figure 3:**
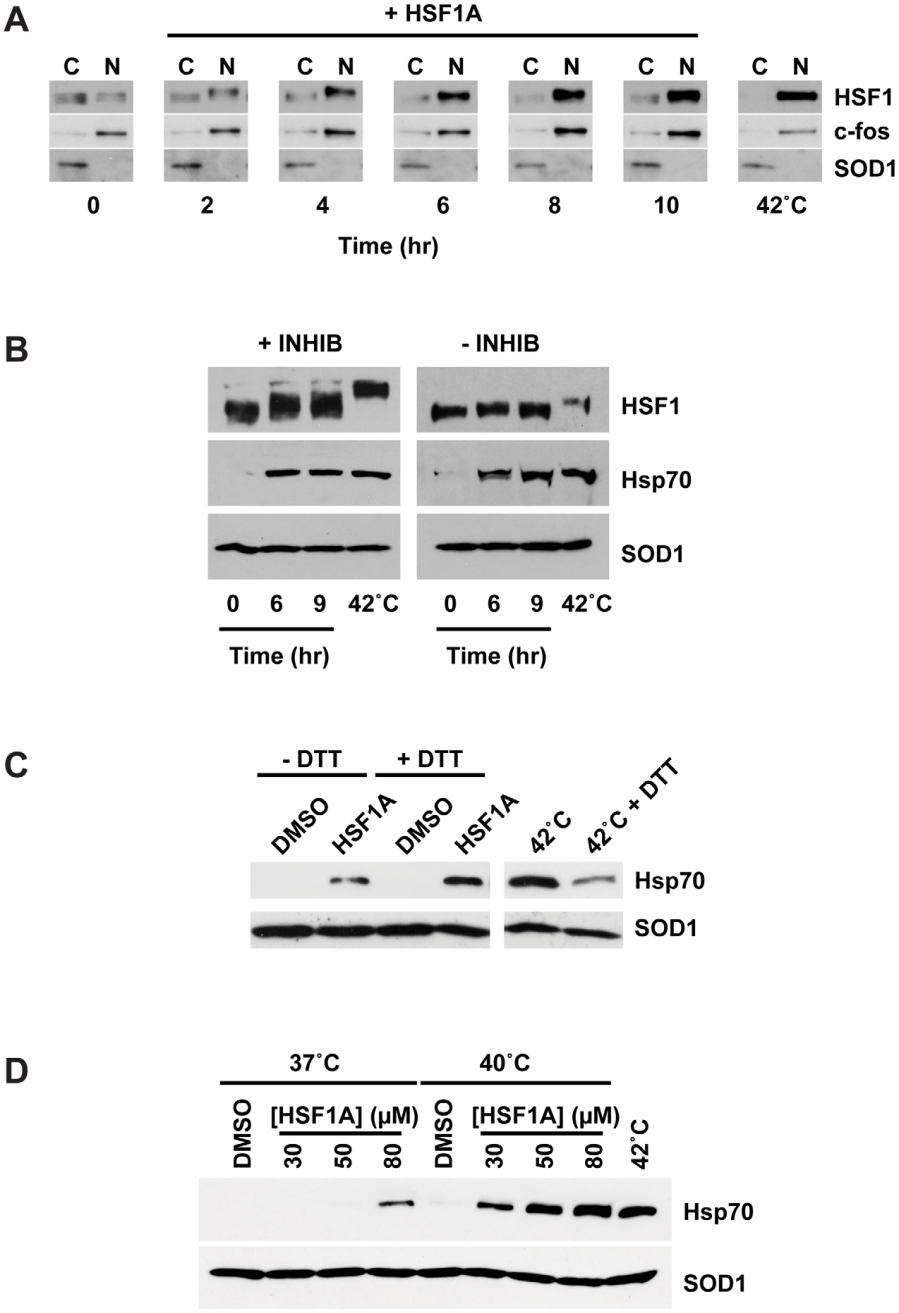
Features of HSF1A-dependent HSF1 activation. (A) HSF1^+/+^ MEFs were treated with 80 µM HSF1A for the indicated time in hours or heat shocked at 42°C for 2 h, and nuclear and cytoplasmic fractions were analyzed for HSF1 by immunoblotting. c-fos and SOD1 serve as nuclear (N) and cytoplasmic (C) markers, respectively. (B) HSF1^+/+^ MEFs were treated with HSF1A for 6 h or 9 h or heat shocked at 42°C for 2 h in the presence (+) or absence (−) of phosphatase inhibitors (INHIB). (C) HSF1^+/+^ MEFs were pretreated with 250 µM DTT for 1 h prior to the addition of 80 µM HSF1A for 15 h or a 2-h heat shock at 42°C followed by a 15-h recovery. For comparison purposes, the heat-shocked samples are shown at a lower exposure than the HSF1A-treated samples. (D) HSF1^+/+^ MEFs were treated with either DMSO or HSF1A (30, 50, or 80 µM) for 15 h at 37°C. For synergistic activation of Hsp70 expression, wild-type MEFs were treated with either DMSO or HSF1A (30, 50, or 80 µM) for 1 h at 37°C prior to a 1-h heat shock at 40°C followed by a 15-h recovery period at 37°C. For control purposes, Hsp70 expression following a 1-h heat shock at 42°C, and a 15-h recovery is shown. SOD1 serves as the protein loading control.

Previous studies demonstrated that in response to proteotoxic stress mammalian HSF1 is activated in a multistep process that involves multimerization, nuclear accumulation, hyperphosphorylation, and DNA binding to promoter HSEs to activate protein chaperone gene expression [19]–[21],[28]. To gain insights into how HSF1A promotes HSF1 activation, the subcellular localization and phosphorylation status of HSF1 was assessed in MEFs treated with HSF1A. Although under control conditions the majority of HSF1 was localized to the cytoplasmic fraction with the Cu, Zn superoxide dismutase control, within 2 h after HSF1A treatment, additional HSF1 was found in the nuclear fraction (c-fos as control), and by 6 h and beyond, the majority of HSF1 was nuclear (Figure 3A). As previously established, an acute heat shock for 2 h resulted in the HSF1 being nearly quantitatively nuclear. HSF1 that was accumulated in the nucleus in response to HSF1A treatment had a reduced electrophoretic mobility in SDS-PAGE as compared to that found in the cytosolic fraction, consistent with possible hyperphosphorylation that is observed to occur in response to an acute heat shock [42],[43]. To explore whether HSF1A treatment results in HSF1 hyperphosphorylation, extracts were prepared from both HSF1A-treated and heat-shocked cells in the presence or absence of protein phosphatase inhibitors. As shown in Figure 3B, extracts from cells incubated with HSF1A or subjected to a 42°C heat shock and prepared in the presence of phosphatase inhibitors showed an HSF1A-dependent reduction in electrophoretic mobility that was not present when extracts were prepared in the absence of phosphatase inhibitors. These results are consistent with HSF1A stimulating HSF1 phosphorylation. Although the phosphatase inhibitor-dependent shift in HSF1 electrophoretic mobility was not as dramatic as that for heat shock treatment, HSF1 phosphorylation occurred in response to both treatments in parallel with expression of the HSF1 target, Hsp70 (Figure 3B).

Previous reports have demonstrated that heat shock– or oxidative stress–mediated activation of HSF1 in vivo or in vitro can be abrogated by treatment with reducing agents such as DTT [44],[45]. Although HSF1A-mediated activation of human HSF1-dependent yeast cells growth is not inhibited by DTT (Figure S4), the ability of DTT to inhibit HSF1 activation by HSF1A was tested in mammalian cells. DTT strongly inhibited heat shock-induced expression of Hsp70, but had no inhibitory effect on HSF1A-dependent activation of Hsp70 expression (Figure 3C). These observations provide additional evidence that HSF1A activates HSF1 in a manner that is mechanistically distinct from a proteotoxic stress.

Previous reports suggest that conditions or small molecules that activate or coactivate HSF1 can act synergistically with heat stress in enhancing protein chaperone expression [31],[46],[47]. The ability of HSF1A to activate protein chaperone expression at subthreshold HSF1A concentrations, in combination with subthreshold temperatures, was assessed by immunoblotting. HSF1A concentrations insufficient to activate HSF1 at 37°C (30 µM and 50 µM) were evaluated at a mild temperature increase (40°C). As shown in Figure 3D, although 30 µM and 50 µM HSF1A are insufficient to activate Hsp70 expression at 37°C, 80 µM HSF1A significantly activated Hsp70 expression at this temperature. Moreover, a 1-h heat shock at 42°C, but not 40°C, followed by a 15-h recovery, was able to activate Hsp70 expression. However, MEFs treated with 30 µM or 50 µM HSF1A for 1 h prior to a 1-h heat shock at 40°C strongly activated Hsp70 expression, and expression in response to 80 µM HSF1A was higher at 42°C. Taken together, these observations indicate that low concentrations of HSF1A can function in concert with modest temperature increases to elevate expression levels of mammalian chaperone proteins.

### HSF1A Reduces PolyQ Aggregation and Cytotoxicity in Neuronal Precursor Cells

Elevated expression of protein chaperones has been shown to reduce cytotoxicity associated with a number of cellular or metazoan models of protein conformational diseases [5]–[13],[48]. In many instances, this is accompanied by a reduction in protein aggregates, supporting the notion that protein aggregation is linked, at least in part, to cellular toxicity [5],[7],[13],[48]. Other reports suggest that reduction of protein aggregates is not essential for the therapeutic effects of protein chaperones, as some studies have shown a reduction in cytotoxicity without an apparent change in protein aggregates [6],[8],[10],[11]. Since metazoan protein chaperones stabilize misfolded proteins rather than disaggregate them [28], any reductions in protein aggregates that have been observed in response to protein chaperone overexpression are likely derived from the ability of protein chaperones to stabilize the misfolded oligomeric precursors [14],[15]. However, analysis of protein aggregate formation still serves as a useful tool for analyzing protein chaperone function because aggregate formation is directly dependent on the folding state of the oligomeric precursor [17].

**Figure 4:**
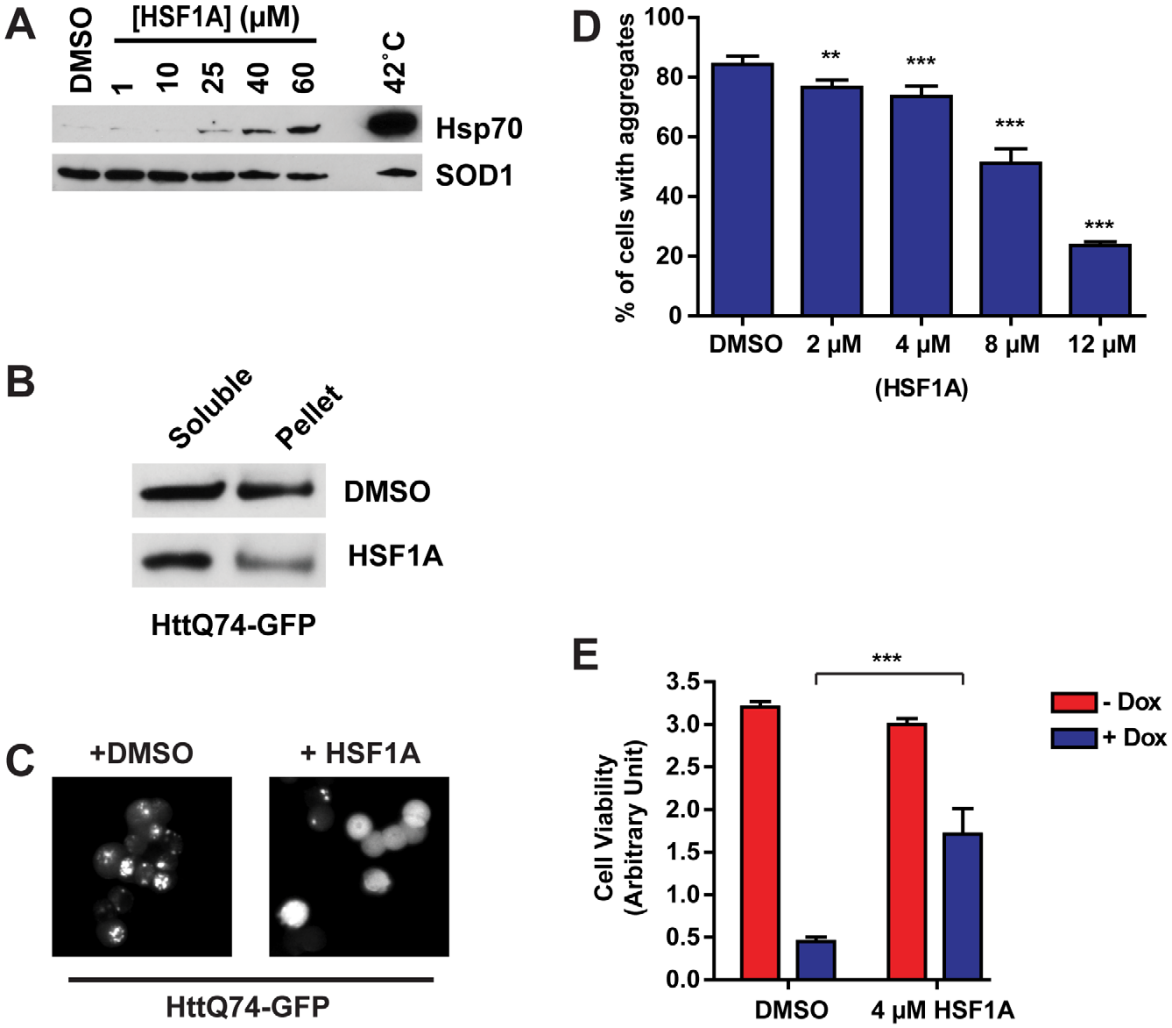
HSF1A suppresses aggregation and cytotoxicity in a cell culture model of Huntington disease. (A) HD-Q74 PC12 cells were incubated with increasing concentrations of HSF1A for 15 h or heat shocked for 2 h at 42°C, followed by a 15-h recovery and Hsp70 levels analyzed by immunoblotting, with SOD1 as a loading control. (B) PC12 cells were pretreated with either DMSO or 10 µM HSF1A for 15 h and doxycycline added to 1 µg/ml, with further incubation for 48 h. Equal amounts of the soluble and insoluble fractions were analyzed for the presence of httQ74-GFP by immunoblotting with anti-GFP antibody. (C) Fluorescence pattern for httQ74-GFP analyzed microscopically in cells pretreated with DMSO or HSF1A prior to induction of httQ74-GFP expression. (D) Quantification of (C) by counting the number of cells containing aggregates expressed as a percentage of the total number of cells counted. For each treatment, approximately 800 HD-Q74 PC12 cells were evaluated. **p<0.01; ***p<0.001. (E) PC12 cells were pretreated with 4 µM HSF1A for 15 h, before the addition of doxycycline (Dox) to 1 µg/ml followed by a 5-d incubation. Cell viability was assayed by the XTT viability assay. ***p<0.001.

A cell culture model of Huntington disease was used determine whether the ability of HSF1A to promote protein chaperone expression can reduce the formation of protein aggregates and the cytotoxicity associated with polyQ protein overexpression [49]. In this model, exon 1 of human *Huntingtin* (htt) containing 74 glutamines fused to green fluorescent protein (GFP) (httQ74-GFP) is expressed in rat phaeochromocytoma (PC12) cells, a rat neuronal precursor cell line, via a doxycycline-inducible promoter. As shown in Figure 4A, HSF1A promoted Hsp70 expression in PC12 cells, detectable by immunoblotting, at 25 µM, a concentration almost 3-fold lower than that required to detect Hsp70 expression by immunoblotting in MEF cells (Figure 2A). In addition, ELISA analysis showed that chronic exposure of PC12 cells to HSF1A at concentrations less than 25 µM promoted Hsp70 activation (Figure S3). To ascertain whether HSF1A pretreatment could ameliorate protein aggregation, cells were pretreated for 15 h with either DMSO or 10 µM HSF1A prior to a 48-h induction of httQ74-GFP expression. HttQ74-GFP aggregates, which are resistant to SDS solubilization, were fractionated from soluble proteins by centrifugation, and both soluble and insoluble protein fractions assayed for abundance of the httQ74-GFP by solubilization with urea-SDS, followed by immunoblotting (Figure 4B). Extracts from cells pretreated with DMSO displayed approximately 50% of the HttQ74-GFP protein in the insoluble fraction. However, significantly less httQ74-GFP was detected in the insoluble fraction when PC12 cells were pretreated with 10 µM HSF1A prior to the induction of httQ74-GFP, suggesting that HSF1A can reduce the aggregation potential of this polyQ protein. Moreover, as previously reported, microscopic evaluation demonstrated that overexpression of httQ74-GFP resulted in the formation of large protein aggregates in approximately 80% of these cells, and this was observed when the cells were pretreated with DMSO (Figure 4C). However, when this neuronal precursor cell line was pretreated for 15 h with 10 µM HSF1A before httQ74-GFP expression, most cells showed diffuse, pan-cellular GFP staining and the absence of visible protein aggregates. Quantification of these results, by counting the number of cell containing aggregates as a function of the total number of cells, revealed that at HSF1A concentrations as low as 2 µM, a reduced number of aggregate-containing cells were observed (Figure 4D). The fraction of cells containing aggregates continued to decrease in a dose-dependent manner such that pretreatment with 12 µM HSF1A resulted in ∼20% of the cells exhibiting aggregates visible by fluorescence microscopy. When PC12 cells were treated with HSF1A after protein aggregates had formed, no reduction in aggregate abundance was observed (unpublished data), supporting the notion that the protein chaperones induced by HSF1A treatment act on the oligomeric precursors rather than the larger aggregates.

We tested whether the ability of HSF1A to reduce protein aggregation might also reduce the cytotoxicity associated with polyQ protein overexpression, as previous reports have shown that prolonged expression of httQ74-GFP results in a large percentage of the cell population undergoing apoptosis [49]. Cells were pretreated with 4 µM HSF1A, a concentration capable of reducing the fraction of aggregate-containing cells by approximately 10% (Figure 4D), for 15 h prior to expression of httQ74-GFP for 5 d. Cells pretreated with DMSO experienced a 6-fold reduction in cell viability, whereas viability was significantly maintained when cells were pretreated with 4 µM HSF1A prior to httQ74-GFP induction (Figure 4E). Together, these results demonstrate that exposure of cultured mammalian neuronal precursor cells to HSF1A activates expression of Hsp70 and simultaneously reduces polyQ protein aggregation and the ensuing cell death.

### HSF1A Ameliorates PolyQ-Induced Cytotoxicity in a Fly Model of Protein Conformational Disease

**Figure 5:**
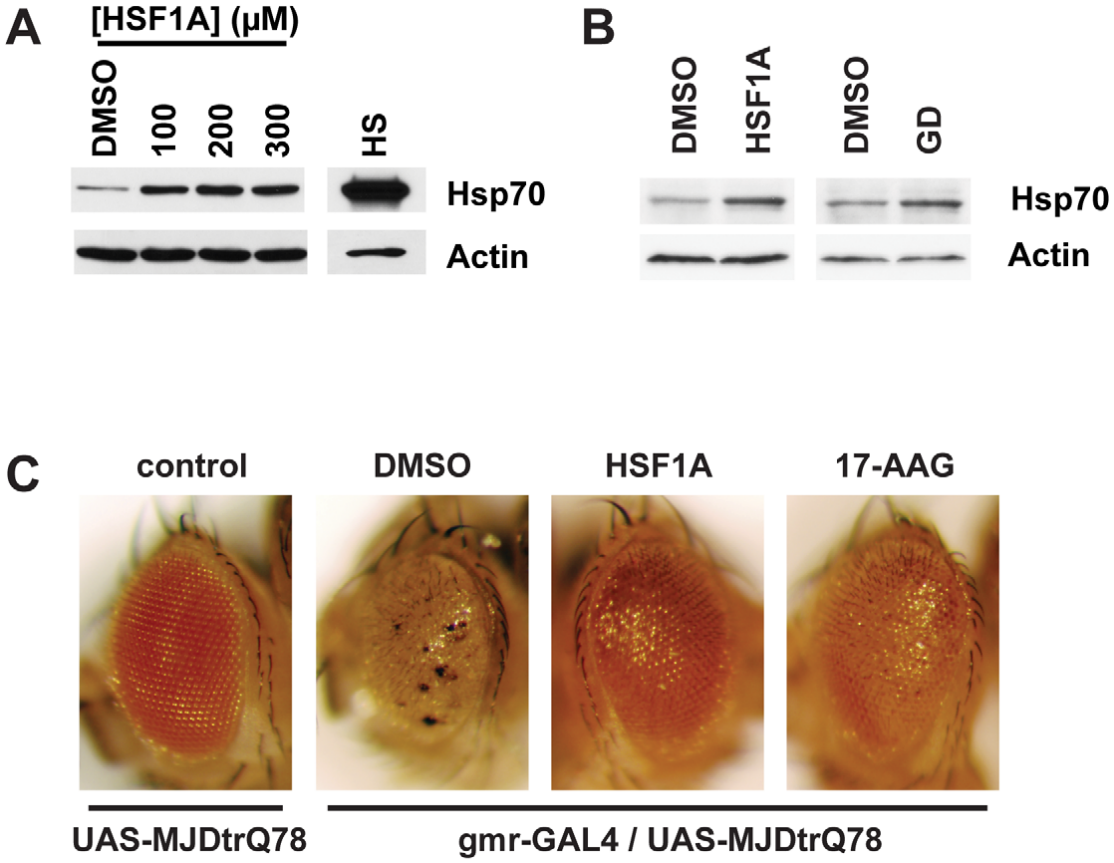
HSF1A promotes Hsp70 expression and reduces polyQ toxicity in fruit flies. (A) *Drosophila* S2 cells were treated with increasing concentrations of HSF1A, or heat shocked (HS) at 37°C for 1 h. Hsp70 expression was analyzed by immunoblotting, with actin as a loading control. (B) W^1118^ flies were raised on food supplemented with DMSO, 5 mM HSF1A, or 0.15 mM geldanamycin (GD) for 3 d. Total protein was extracted from flies and analyzed for Hsp70 expression by immunoblotting as for (A). (C) UAS-MJDtrQ78 flies were crossed to gmr-GAL4 flies in the chronic presence of food supplemented with DMSO, 400 µM HSF1A, or 5 µM 17-AAG. Reductions in eye morphological defects and depigmentation, caused by polyQ-protein expression, are observed with HSF1A and 17-AAG treatment. The lowest effective concentration of HSF1A at which reductions in defects was observed was 400 µM. Control flies are UAS-MJDtrQ78 flies lacking the Gal4 transcription factor. These data are representative of three independent experiments.

The fruit fly *Drosophila melanogaster* has been used as an elegant metazoan model of human neurodegenerative diseases that include Huntington, Machado-Joseph disease (MJD) and Parkinson disease [6],[50],[51]. Although the yeast and mammalian HSF1 proteins are not highly structurally conserved, *Drosophila* HSF shares strong structural conservation and is regulated via steps that are similar to that of mammalian HSF1 [19],[21]. Cultured *Drosophila* S2 cells were used to test whether HSF1A is capable of promoting Hsp70 expression, indicative of *Drosophila* HSF1 (dHSF1) activation. Treatment of S2 cells with a range of HSF1A concentrations for 15 h strongly activated the expression of Hsp70 (Figure 5A). To determine whether HSF1A could promote Hsp70 expression in whole animals, W^1118^ flies were maintained on food supplemented with DMSO, 5 mM HSF1A, or as a positive control for HSF activation via Hsp90 inhibition, 0.15 mM geldanamycin for 3 d. Immunoblotting for Hsp70 expression from total body protein extracts indicated that both HSF1A as well as geldanamycin [52] induced expression of Hsp70 in vivo (Figure 5B).

We utilized a *Drosophila* model of MJD to ascertain whether HSF1A could reduce polyQ-mediated cytotoxicity in vivo. In this strain, a truncated form of the human MJD/SCA3 protein, containing 78 glutamines (MJDtrQ78), is expressed under the control of the UAS promoter [51]. UAS-MJDtrQ78 flies were crossed to flies expressing the UAS-specific Gal4 transcription factor under control of the eye-specific *gmr* driver. Due to the eye-specific expression of MJDtrQ78, cytotoxicity is manifested as disruption of eye morphology, depigmentation, and reduction in eye size and is observed in the progeny almost immediately after eclosion [51]. UAS-MJDtrQ78 flies were crossed while being maintained on food supplemented with either DMSO, HSF1A, or 17-AAG, a potent Hsp90 inhibitor that was previously demonstrated to ameliorate polyQ expression phenotypes in this fly model [53]. After eclosion, flies were maintained on supplemented food for approximately 24 h, and eye phenotypes were inspected microscopically. Similar to previous reports [51], a severe distortion in eye morphology and depigmentation were observed in the DMSO-treated flies (Figure 5C). However, flies fed 400 µM HSF1A or 17-AAG exhibited a significant reduction in the phenotypes induced by MJDtrQ78 overexpression, with eye morphology, eye size, and pigment color resembling that observed in control animals (Figure 5C). HSF1A treatment was unable to ameliorate MJDtrQ78-dependent phenotypes at a nonpermissive temperature in flies expressing a temperature-sensitive allele of HSF1 [54], supporting the notion that HSF1A promotes its beneficial effects through HSF1 (Figure S5). Together, these results strongly suggest that HSF1A promotes HSF1-dependent protein chaperone expression in metazoans and partially suppresses cytotoxicity due to polyQ-protein expression.

### HSF1A Does Not Bind Hsp90

**Figure 6:**
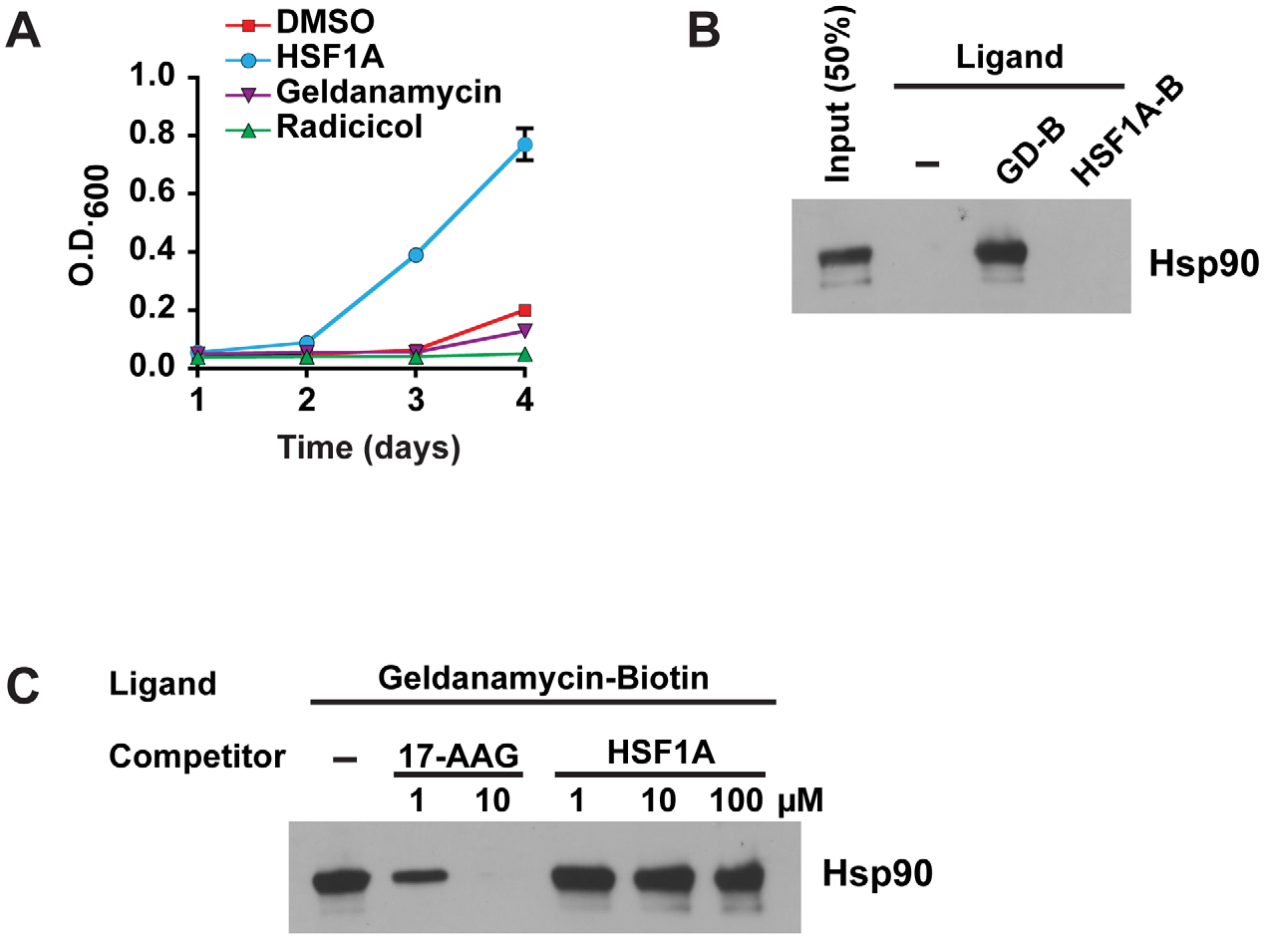
HSF1A is unlikely to be an inhibitor of Hsp90. (A) Yeast cells were treated with either 10 µM HSF1A, 10 µM geldanamycin, or 10 µM radicicol and growth was assessed as in Figure 1C. (B) Purified Hsp90α was incubated with increasing concentrations of 17-AAG or HSF1A for 30 min at 4°C and then incubated with 1 µM geldanamycin-biotin for 1 h at 4°C. (C) Geldanamycin-biotin–bound Hsp90 was captured using neutravidin-agarose beads and analyzed by immunoblot analysis. Hsp90α was incubated with either 10 µM geldanamycin-biotin (GD-B) or 100 µM HSF1A-biotin (HSF1A-B) and was analyzed as described in Figure 2B.

Previous studies suggest that Hsp90 and additional cochaperones exist in a heteroprotein complex that, in addition to their central role in cellular signaling, function to repress HSF1 in the absence of stress. In response to proteotoxic stress or pharmacological inhibitors of Hsp90, this complex dissociates, resulting in the multimerization of HSF1 [23]. As such, we reasoned that the inability of human HSF1 to be activated in yeast might stem from a repressive interaction between yeast Hsp90 and human HSF1 and that HSF1A promotes HSF1 activation in yeast by disrupting this interaction. To test this hypothesis, the efficacy of the potent Hsp90 inhibitors geldanamycin and radicicol, in promoting human HSF1-dependent yeast growth was evaluated. Exposure to either 10 µM geldanamycin or 10 µM radicicol for 3 h activated expression of the yeast HSF-dependent *SSA3-lacZ* reporter gene, reflective of their previously established function as Hsp90 inhibitors in yeast [55] (Figure S6). However, neither geldanamycin nor radicicol were able to promote human HSF1-dependent yeast growth under the same conditions in which they are potent Hsp90 inhibitors (Figure 6A), suggesting that HSF1A is unlikely to act as an Hsp90 inhibitor. Consistent with this notion, HSF1 was not activated in a yeast strain that expresses ∼5% of the wild-type levels of Hsp90 [56] (unpublished data).

Many pharmacological inhibitors of Hsp90 such as geldanamycin and 17-AAG target the amino-terminal ATP binding pocket of Hsp90, thereby inhibiting its chaperone function [57]. To test whether HSF1A has affinity for the Hsp90 ATP-binding pocket, we performed competitive binding assays in vitro using a biotinylated geldanamycin (GD-B) molecule. As shown in Figure 6B, the high-affinity Hsp90 inhibitor 17-AAG was able to compete with GD-B for Hsp90 binding at 1 µM and 10 µM. In contrast, HSF1A, even at concentrations 100-fold higher than GD-B, was unable to compete for Hsp90 binding (Figure 6B), suggesting that HSF1A does not bind the ATP-binding pocket of Hsp90. Because other small molecule inhibitors of Hsp90, including celastrol, novobiocin, and EGCG, are thought to bind Hsp90 at the carboxy-terminus [57], we ascertained whether HSF1A could bind to Hsp90 at a region outside of the ATP-binding pocket. To assay for HSF1A binding to Hsp90, we generated an HSF1A-biotin conjugate (HSF1A-B) (Figure S7B) and assayed the ability of HSF1A-B to interact with Hsp90. Although GD-B readily interacted with Hsp90 (Figure 6C), HSF1A-B, assayed at a concentration 10-fold higher than GD-B, did not interact with Hsp90. Furthermore, HSF1A-B did not interact with Hsp90 or the cochaperones Cdc37, Hop, p23, or Hsp70, nor HSF1 itself in pull-down experiments with mammalian cell extracts (unpublished data). Given that geldanamycin and radicicol cannot promote human HSF1-dependent yeast growth and that HSF1A does not interact with Hsp90, together these data strongly suggest that HSF1A is not acting as an Hsp90 inhibitor in activating HSF1 in yeast or mammalian cells.

### HSF1A-Biotin Copurifies with the TRiC/CCT Complex

**Figure 7:**
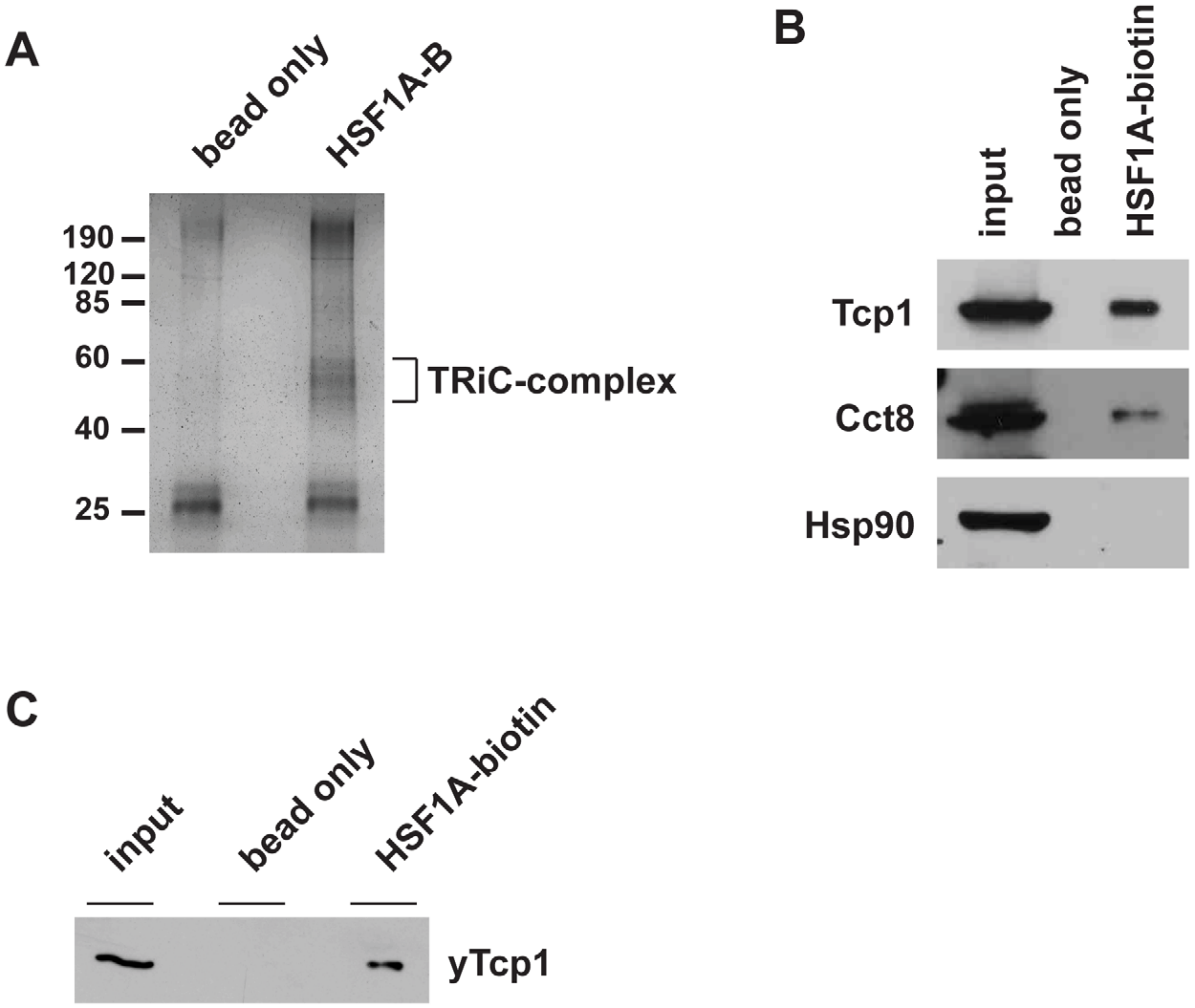
HSF1A-biotin associates with the TRiC/CCT complex. (A) Cell extracts from mouse embryonic fibroblasts were incubated with 100 µM HSF1A-biotin, and associated proteins were then purified with neutravidin-agarose beads. HSF1A-biotin-interacting proteins were resolved by SDS-PAGE and visualized by silver staining. (B) HSF1A-biotin-interacting proteins were purified as in (A) from HeLa cell extracts and analyzed by immunoblot analysis for Tcp1, Cct8, and Hsp90. (C) HSF1A-biotin-interacting proteins were purified as in (A) from yeast cell extracts and analyzed by immunoblot analysis for yTcp1.

In order to identify potential targets of HSF1A, we utilized the HSF1A-biotin conjugate to identify proteins that associated with HSF1A in whole-cell extracts generated from MEF cells. SDS-PAGE in conjunction with silver staining revealed that several proteins between 50–60 kDa in size consistently copurified with HSF1A-B (Figure 7A). Similar-sized proteins were also found to associate with HSF1A-B in protein extracts generated from HeLa, human APRE-19, as well as yeast cells (unpublished data), suggesting that the association of HSF1A-B with these proteins is not cell specific. To identify HSF1A-B-interacting proteins, protein bands were excised from the gel, trypsinized to generate peptides, and then subjected to tandem mass spectrometry (MS). Analysis of the liquid chromatography tandem mass spectrometry (LC-MS/MS) data using the Mascot database identified these proteins as all eight subunits of the TRiC/CCT cytosolic chaperone complex [58]. These results were confirmed in part by immunoblot analysis showing that HSF1A-B copurified with Tcp1/Cct1 and Cct8 in mammalian cell extracts (Figure 7B) as well as yTcp1 in yeast cell extracts (Figure 7C).

### HSF1A Activates Human HSF1, but Not Yeast HSF

The TRiC/CCT complex is required for the accurate folding of cytoskeletal components including tubulin and actin as well as most WD40 repeat-containing proteins [58]. As such, it is possible that an interaction between HSF1A and TRiC might disrupt TRiC function thereby promoting accumulation of misfolded proteins and activation of HSF1 activity. To test whether HSF1A promotes the accumulation of unfolded proteins, we assessed the ability of HSF1A to activate the *SSA3*-*LacZ* reporter gene in yeast since its activation is dependent on the stress-induced activation of yeast HSF in response to the accumulation of unfolded proteins [59]. Although a 3-h heat shock at 39°C activated expression of the *SSA3-lacZ* reporter gene, no activation of this reporter was detected after treatment with 20 µM HSF1A (Figure S8), a concentration sufficient to promote human HSF1-dependent growth in strain DNY75 (Figure 1C). The inability of HSF1A to activate yeast HSF strongly suggests that HSF1A does not promote human HSF1 activation in yeast by promoting the accumulation of unfolded proteins but instead acts on structures or regulatory mechanisms unique to human HSF1, potentially through modulation of the TRiC complex. In support of this notion, human HSF1 is not activated in yeast by several compounds that enhance protein chaperone expression in yeast and mammalian cells by promoting the accumulation of damaged and misfolded proteins [60] (Figure S9).

## Discussion

Here, we describe a novel humanized yeast high-throughput screen for the identification of small molecule activators of human HSF1. Utilizing this screen, we have identified HSF1A, a molecule capable of promoting human HSF1-dependent yeast cell growth, HSF1 multimerization in yeast, as well as HSF1-dependent protein chaperone expression in mammalian cell culture and in fruit flies. Human HSF1 is normally activated by cellular stress conditions that cause proteotoxicity through protein damage. As such, previous screens for small molecule activators of HSF1 in mammalian cells have identified molecules that elevate protein chaperone expression through the imposition of proteotoxic stress conditions, such as direct protein thiol oxidation [34], thereby disrupting protein homeostasis and resulting in protein damage. In addition, since HSF1 is repressed through an interaction with Hsp90, small molecule inhibitors of Hsp90 are often identified as HSF1-activating molecules [33].

Although the precise mechanism by which HSF1A activates human HSF1 is not yet understood, several observations suggest that HSF1A does not activate HSF1 through the inhibition of Hsp90 activity nor through the imposition of a proteotoxic stress. The inability of well-established inhibitors of both mammalian and yeast Hsp90, geldanamycin and radicicol, to promote HSF1-dependent yeast growth suggests that HSF1A is promoting HSF1 activation in yeast independent of Hsp90 inhibition. This is confirmed by our data showing that HSF1A does not bind to Hsp90 and does not efficiently compete with geldanamycin for Hsp90 binding. In addition, HSF1A was not able to promote yeast HSF activation, also thought to be repressed by Hsp90 [61],[62]. Moreover, unlike the sensitivity of acute heat shock or celastrol treatment to DTT, HSF1A activation of HSF1 was not inhibited by this exogenous thiol reductant, suggesting that HSF1A does not promote HSF1 activation by inducing thiol oxidation and protein damage.

Interestingly, our data show that HSF1A interacts with the TRiC/CCT complex in both yeast and mammalian cell extracts, though it remains unclear whether this interaction is direct or indirect. In addition, it remains unclear whether or not HSF1A affects TRiC activity. To date, no known role for TRiC in HSF1 regulation has been described. As such, further investigation is required to ascertain whether HSF1A modulates TRiC activity and how this might impinge on HSF1 activation. Interestingly, TRiC chaperone activity was recently shown to be a potent negative regulator of polyQ protein aggregation and cytotoxicity in yeast and mammalian systems [48],[63]. Since HSF1A similarly ameliorated polyQ aggregation and cytotoxicity, our data are consistent with a model in which HSF1A might act as a positive regulator of TRiC activity.

Strong evidence in cellular and animal model systems of neurodegenerative diseases associated with protein misfolding support the idea that protein chaperones act to ameliorate the biochemical and neurodegenerative phenotypes [5]–[13],[48]. Although elevated expression of Hsp70 or Hsp40 can significantly suppress protein aggregation, coexpression of the two chaperones can synergize the suppression of protein aggregation, enhance protein solubility, and ameliorate neuronal loss as a consequence of polyQ protein or α-synuclein expression [8],[9]. Moreover, a constitutively active form of HSF1 suppressed polyQ aggregation in cultured cells and in mice, and enhanced the longevity of a mouse model of polyQ-based Huntington disease [5]. Given that HSF1 coordinately activates the expression of multiple protein chaperones and other cytoprotective genes [64]–[67], HSF1 activation could be of potential therapeutic value in neurodegenerative diseases associated with a wide array of manifestations of protein misfolding.

Here, we demonstrate in cultured neuronal precursor cells, and in a fruit fly model of polyQ disease, that HSF1A can suppress the aggregation of a polyQ protein and cytotoxicity associated with polyQ expression. Interestingly, in both the cell culture and fly polyQ model, the concentration of HSF1A required to reduce cytotoxicity is significantly less than the concentration required for maximum Hsp70 expression. Similar results have been described for the Hsp90 inhibitor geldanamycin, which was able to rescue cell viability in a *Drosophila* model of Parkinson disease at concentrations significantly lower than that required to induce Hsp70 expression maximally [52],[68]. Together, these results suggest that relatively small yet chronic increases in protein chaperone expression are sufficient to significantly stabilize misfolded polyQ proteins and thereby reduce cytotoxicity. It is also possible that low levels of HSF1A reduce the threshold of HSF1 activation in response to the accumulation of unfolded proteins at physiological temperatures. This hypothesis is supported by our results demonstrating that HSF1A can reduce the temperature threshold required for HSF1 activation. Previous studies have shown that misfolded polyQ proteins are turned over by autophagy and ubiquitin-dependent proteosomal degradation and that protein chaperones can impinge on both processes [69],[70]. In addition, HSF1 and protein chaperones have been suggested to be potentiators of oncogenic transformation [71]. Although we do not yet fully understand whether HSF1A-mediated protein chaperone expression affects these processes, these hypotheses are currently under investigation. Given the broad range of protein chaperone functions, the humanized yeast-based screening results described here present a new avenue for the identification of new classes of small molecules that could have therapeutic efficacy, via potentially distinct mechanisms, in neurodegenerative diseases caused by protein conformational disorders.

## Materials and Methods

### Yeast Screen for Human HSF1 Activators

To screen for small molecule activators of human HSF1 in yeast, we generated yeast strain DNY75 that expresses yeast HSF under control of the galactose-inducible and glucose-repressible *GAL1* promoter and human HSF1 under the control of the constitutive GPD promoter. Yeast cultures were grown overnight to mid-log phase in SC-URA-TRP media containing 2% raffinose and 0.01% galactose. Cultures were reinoculated to an optical density at 600 nm (OD_600_) = 0.0005 in SC-URA-TRP containing 4% dextrose to extinguish expression of yeast HSF. Using a Beckman Biomek FX robot, 200 µl of the yeast culture were pipetted into each well of a 96-well plate and supplemented with compounds (10 µM final concentration) from a combinatorial compound library (PPD Discovery) containing 10,440 compounds [40] or DMSO as solvent at the same final percent volume. Cells were incubated at 30°C for 4 d and their growth monitored spectrophotometrically at OD_600_ using a Spectra Max 384 plate reader (Molecular Devices). Chemicals promoting yeast cell growth were selected from the library and further validated in two additional rounds of screening. In total, we identified 33 positive-hit molecules (0.32% hit rate) able to promote human HSF1-dependent yeast growth. Growth curve experiments were carried out in 96-well plates. Data shown for all growth curves are averages of four independent experiments with associated standard deviations. High-throughput screening (HTS) assays are usually assessed for their suitability using a statistical Z′-factor by comparing positive and negative controls [72]. However, during the development of this assay, we were unable to calculate a Z′-factor to assess this assay since a positive control molecule able to promote human HSF1-dependent growth in yeast was unavailable. Upon the identification of HSF1A, we reassessed the Z′-factor using the formula Z′ = 1−[(3σ_c+_+3σ_c−_)/(|μ_c+_−μ_c−_|)], where σ = standard deviation, μ = mean, c+ = HSF1A, and c− = DMSO of the growth rate of the yeast culture between day 1 to day 4. The Z′-factor was calculated to be 0.51, indicating that this assay is appropriate for further HTS studies.

### Yeast Strains

Strain DNY75 was derived from PS145 (*MATa ade2 trp1 leu2 his3 ura3 hsf1Δ::LEU2 Ycp50gal-yHSF1*) [43] by deleting *PDR5*, *SNQ2*, and *ERG6* in successive steps using a *loxP-KanMX4-loxP* deletion cassette. After deletion of *PDR5* and *SNQ2*, the *KanMX4* gene was removed by recombination via expression of the Cre-recombinase-expressing plasmid pSH47 [73]. The *KanMX4* gene was not removed after the disruption of the *ERG6* gene. For screening of hHSF1-activating molecules, DNY75 was transformed with pRS424-GPD-hHSF1 by electroporation. DNY227 was derived from DNY75 by exchanging the inducible plasmid Ycp50GAL-yHSF1 with the constitutively expressed pRS314-yHSF1.

### Hsp90 Binding Assays

Purified Hsp90α (2 µg, Assay Designs) in Hsp90 binding buffer (10 mM Tris, 50 mM KCl, 5 mM MgCl_2_, 20 mM NaMoO_4_, 0.01% NP40) was incubated with either GD-biotin (Biomol) or HSF1A-biotin for 1 h at 4°C, and bound Hsp90 was then captured by incubating with neutravidin-agarose beads for 30 min at 4°C. The beads were washed three times in Hsp90 binding buffer, and Hsp90 was eluted from the beads by incubating in Laemmli Sample Buffer at 95°C for 5 min. For competition experiments, Hsp90α was pre-incubated with either 17-AAG or HSF1A for 30 min at 4°C prior to the addition of GD-biotin.

### Identification of HSF1A-Biotin-Associated Proteins

Protein extracts were generated from mammalian and yeast cell cultures using biotin binding buffer (20 mM HEPES, 5 mM MgCl_2_, 1 mM EDTA, 100 mM KCl, 0.03% NP-40) supplemented with 1% Triton X-100 and protease inhibitors. A total of 1 mg of a whole-cell extract was incubated with 100 µM HSF1A-biotin for 15 h at 4°C. HSF1A-biotin-associated proteins were captured by incubating with neutravidin-agarose beads for 90 min at 4°C. The beads were washed three times in biotin binding buffer and the associated proteins were eluted from the beads by incubating in Laemmli Sample Buffer at 95°C for 5 min. Proteins that co-purified with HSF1A-biotin were resolved on a 4–20% SDS-PAGE gel and visualized by colloidal blue or silver staining. Protein bands were excised from the gel, and the gel slice was subjected to in-gel digestion (detailed protocol at http://www.genome.duke.edu/cores/proteomics/sample-preparation/), followed by LC-MS/MS analysis using a nanoAcquity liquid chromatograph and a QToF Premier mass spectrometer (Waters Corp). The top three most intense multiply-charged ions from each MS scan were interrogated by tandem MS. Raw data were processed using Mascot Distiller v2.0 and searched against the SwissProt database with *Mus musculus* taxonomy (v57.4, http://www.expasy.org) using Mascot v2.2 database search engine, with 20 ppm precursor and 0.04 Da product ion tolerance. Iodoacetamide derivative of cysteine (fixed) and oxidation of methionine (variable) were specified in the Mascot search. Scaffold (version 2.05.02, http://www.proteomesoftware.com) was used to validate MS/MS-based peptide and protein identifications. Peptide identifications were accepted if they could be established at greater than 80.0% probability as specified by the Peptide Prophet algorithm. Protein identifications were accepted if they could be established at greater than 95.0% probability and contained at least two identified peptides.

### Cell Culture

Mammalian cell lines used in this study were HSF1^+/+^ and *hsf1^−/−^* MEF cells [74], HeLa cells, and inducible PC12 cells expressing httQ74-GFP [49]. For a *Drosophila* cell line, we used S2 cells. HSF1^+/+^ and *hsf1^−/−^* MEFs used in this study have been described elsewhere [41]. MEFs were maintained on DMEM supplemented with 10% fetal bovine serum (FBS), 0.1 mM nonessential amino acids, 100 U/ml penicillin/streptomycin, and 55 µM 2-mercaptoethanol. HD-Q74 PC12 cells were previously described [49] and were maintained on DMEM supplemented with 5% Tet-approved FBS, 10% horse serum, 100 µg/ml G418, 75 µg/ml Hygromycin B, and 100 U/ml penicillin/streptomycin. For all experiments, the cells were washed once in PBS and then shifted with serum-free OPTI-MEM medium (Invitrogen) supplemented with 100 U/ml penicillin/streptomycin, 0.1 mM nonessential amino acids, and 55 µM 2-mercaptoethanol immediately prior to the addition of HSF1A. The cells were maintained in serum-free media throughout the course of experiments. S2 cells were maintained and assayed in Schneider's *Drosophila* medium supplemented with 10% FBS.

### EGS Cross-Linking and Immunoblotting Analysis

Protein extracts were generated from cell cultures using cell lysis buffer (25 mM Tris, 150 mM NaCl, 1% Triton X-100, 0.1% SDS, 1 mM EDTA) supplemented with protease inhibitors. Protein concentrations were quantified using the BCA assay and 10–20 µg of total protein was resolved by SDS-PAGE, transferred to a nitrocellulose membrane, and proteins of interest detected by immunoblot analysis following standard procedures. For HSF1 phosphorylation experiments, protein extracts were isolated either in the presence or absence of the Halt phosphatase inhibitor cocktail (Pierce). For nuclear localization experiments, nuclear and cytoplasmic fractions were isolated using the NE-PER kit (Pierce). HSF1 multimerization state was assessed using the amine-specific cross-linker ethylene glycol bis-succinimidyl succinate (EGS) (Pierce). DNY227 was grown in the presence of DMSO or 20 µM HSF1A for 18 h to a final OD_600_ = 0.8. Cells were washed once with water and extracts prepared by glass bead lysis in HEGNT buffer (20 mM HEPES [pH = 7.5], 1 mM EDTA, 10% glycerol, 0.4 M NaCl, 1% Triton X-100). Protein extracts (50 µg) were incubated with either DMSO or 0.5 mM EGS for 30 min at 24°C. The cross-linking reaction was quenched via the addition of 50 mM glycine/0.025 mM Tris (pH = 7.5) for 15 min at 24°C. Proteins were fractionated through a 7.5% SDS-polyacrylamide gel and analyzed by immunoblotting with a polyclonal antibody specific to human HSF1. Primary antibodies used were anti-Hsp90α (9D2, Assay Designs), anti-Hsc70/Hsp70 (W27, Santa Cruz Biotechnology), anti-Hsp25 (SPA-801, Assay Designs), anti-c-fos (6-2H-2F, Santa Cruz Biotechnology), anti-SOD1 (SOD101, Assay Designs), anti-dHsp70 (dN-12, Santa Cruz Biotechnology), anti-actin (AB-5, BD Transduction), anti-TCP1 (2B2-D6, ABNOVA), anti-CCT8 (B02, ABNOVA), anti-TCP1 (91a, Assay Designs for immunoblot analysis of yeast Tcp1), and anti-GFP (SC-8334, Santa Cruz Biotechnology) used according to the provider's instructions and an affinity-purified rabbit polyclonal anti-HSF1 antibody directed against the HSF1 sequence ISLLTGTEPHKAKDPTVS, which cross reacts with mouse, rat, and human HSF1 was used at a 1∶1,000 dilution.

### RNA Blotting

MEF Cells were seeded into 10-cm plates (3×10^6^ cells/plate) and exposed to either DMSO or HSF1A for 6 h or heat shocked at 42°C for 2 h followed by a 15-h recovery at 37°C. mRNA was isolated from cells using the RNeasy Kit (Qiagen) according to the manufacturer's recommendations. Total RNA (10 µg) was analyzed as described [64], utilizing mHsp70-1 cDNA as a template for probe generation. An oligonucleotide complementary to mouse 18S rRNA was used as a probe for loading.

### β-Galactosidase Assays

For β-galactosidase assays, yeast strain DNY227 transformed with YEp24-SSA3p-LacZ was grown to mid-log phase in SC-URA medium and treated with DMSO or 20 µM HSF1A for 6 h. For comparison, the yeast cells were heat shocked at 39°C for 3 h. β-Galactosidase activity was measured as previously described [75]. Data shown are averages of three independent experiments with associated standard deviations.

### Fluorescence Microscopy

PC12 cells expressing httQ74-GFP were seeded into 6-well glass bottom plates (6×10^5^ cells/well), treated with 10 µM HSF1A or DMSO for 15 h and expression httQ74-GFP induced by the addition of 1 µg/ml doxycycline. Fluorescence was analyzed using a Zeiss Axio Observer fluorescence microscope and images deconvoluted with MetaMorph software. For quantification of fluorescence microscopy analysis, approximately 800 cells were counted for each treatment. The number of cells containing aggregates was calculated as a percentage of the total number of cells counted. The data shown are derived from four independent experiments and are given as averages with associated standard deviations. Statistical significance was calculated with Prism 4 using the unpaired Student *t*-test. **p<0.01; ***p<0.001.

### Cytotoxicity Assays

PC12 cells seeded into a 96-well plate (5×10^4^ cells/well) were treated with increasing concentrations of HSF1A for 15 h, at which time httQ74-GFP expression was stimulated by incubation in the presence of 1 µg/ml doxycycline for 5 d. Cell viability was assessed via the XTT viability assay (Roche) per the manufacturer's recommendations. ***p<0.001.

### Protein Aggregation Analysis

PC12 cells were seeded (5×10^5^ cells/well) into a 6-well plate and treated with either DMSO or 10 µM HSF1A for 15 h, at which time expression of httQ74-GFP was induced via the addition of 1 µg/ml doxycycline followed by a 48 h incubation. Extracts were prepared, and soluble and insoluble fractions were separated by centrifugation and analyzed by immunoblotting as previously described [5].

### *Drosophila* Experiments

The fly stocks gmr-GAL4 and UAS-MJDtr-Q78^strong^ were previously described [51]. All fly crosses were carried out at 25°C in a temperature- and humidity-controlled incubator. Fly food was prepared using Fisher Scientific Jazz Mix *Drosophila* food and supplemented with either DMSO, 400 µM HSF1A, or 5 µM 17-AAG. gmr-GAL4 virgins were crossed to UAS-MJDtr-Q78^strong^ males on supplemented food; all progeny from the crosses carried one copy each of the gmr-GAL4 and UAS-MJDtr-Q78^strong^ transgenes. Progeny were allowed to age for approximately 24 h before imaging, and fly head images were captured using an Olympus SZX7 microscope and DP71 camera. For induction of Hsp70 expression, W^1118^ flies were maintained on food supplemented with either DMSO, 5 mM HSF1A, or 0.15 mM geldanamycin for 3 d. Total protein was extracted from five to ten flies and assayed for Hsp70 expression by immunoblot analysis.

In order to test whether HSF1 was required for HSF1A-dependent amelioration of MJD-induced cytotoxicity the *hsf^4^* allele [54] was recombined onto a chromosome carrying the UAS-MJDtrQ78^strong^ transgene. The recombinant *hsf^4^*, UAS-MJDtrQ78^strong^ flies were then crossed to gmr-Gal4 flies and maintained at 25°C. Growth at 25°C was semipermissive for *hsf^4^* activity, allowing *hsf^4^*-expressing flies to develop similarly to wild-type flies but essentially ablating HSF activity in adult flies [54].

## Supporting Information

Figure S1 **HSF1C activates human HSF1 function in yeast and mammalian cells.** (A) Structure of HSF1C. (B) Yeast cells (DNY75) expressing wild-type human HSF1 were supplemented with 10 µM HSF1A, 10 µM HSF1C or DMSO, and grown in 96-well plates for 4 d. Growth was monitored by measuring OD_600_. (C) HSF1^+/+^ MEFs were treated with DMSO, 100 µM HSF1A, or 100 µM HSF1C for 15 h or heat shocked for 2 h at 42°C followed by a 15-h recovery. Total protein was analyzed for Hsp70 by immunoblotting. GAPDH serves as a loading control.
(0.59 MB TIF)

Figure S2 **HSF1A promotes expression of Hsp70 in human cells.** HeLa cells were treated with increasing concentrations of HSF1A for 15 h or heat shocked for 2 h at 42°C followed by a 15-h recovery. Total protein was extracted and analyzed for Hsp70 expression by immunoblotting. GAPDH serves as a loading control.
(0.46 MB TIF)

Figure S3 **HSF1A promotes Hsp70 expression at low micromolar concentrations.** PC12 cells were incubated with increasing concentrations of HSF1A for 72 h, and Hsp70 concentration was measured as a function of total protein concentration by ELISA (Assay Designs).
(0.18 MB TIF)

Figure S4 **HSF1A-dependent activation of human HSF1 in yeast is not repressed by DTT.** DNY75 cells were treated with 10 µM HSF1A in the absence or presence of 250 µM DTT and grown in 96-well plates for 4 d. Growth was monitored by measuring OD_600_.
(0.26 MB TIF)

Figure S5 **HSF1A does not reduce polyQ toxicity in flies carrying the** ***hsf^4^*** **allele.** *hsf^4^*, *UAS-MJDtrQ78* recombinant flies were crossed to gmr-GAL4 flies in the chronic presence of food supplemented with DMSO or 400 µM HSF1A and maintained at 25°C, a semipermissive temperature for *hsf^4^* activity. No reduction in polyQ-related phenotypes is observed in response to HSF1A treatment, suggesting that full HSF activity is required for HSF1A-dependent amelioration of polyQ induced phenotypes.
(2.01 MB TIF)

Figure S6 **Geldanamycin and radicicol promote activation of SSA3-lacZ.** Yeast strain DNY227, harboring the yHSF1-dependent *SSA3-lacZ* reporter gene, was exposed to DMSO, 10 µM geldanamycin (GD), or 10 µM radicicol for 3 h upon which time reporter gene activation was assessed by β-galactosidase activity assays.
(0.26 MB TIF)

Figure S7 **(A) Structure, ^1^H/^13^C, and EIMS data of HSF1A. (B) Structure, ^1^H/^13^C, and EIMS data of HSF1A-biotin.**
(0.62 MB TIF)

Figure S8 **HSF1A does not activate yeast HSF.** Yeast cells expressing the yHSF1-dependent *SSA3-lacZ* reporter gene were grown at 30°C and exposed to 20 µM HSF1A or DMSO for 6 h or heat shocked at 39°C for 3 h. Reporter gene activation was assessed by β-galactosidase activity assays.
(0.29 MB TIF)

Figure S9 **Human HSF1 is not activated in yeast by proteotoxic agents.** Yeast cells were treated with either HSF1A, resveratrol (RV), azetidine (AZC), TPCK, TLCK, puromycin (PM), or menadione (MD) at a concentration of 10 µM and for 4 d. Growth was monitored by measuring OD_600_. OD_600_ readings at day 4 are shown.
(0.25 MB TIF)

## Abbreviations

HSE: heat shock element
MEF: mouse embryonic fibroblast
MJD: Machado-Joseph disease
MS: mass spectrometry
OD_600_: optical density at 600 nm
