# Supplementary figures and images for: Modulation of Heat Shock Transcription Factor 1 as a Therapeutic Target for Small Molecule Intervention in Neurodegenerative Disease
Source: PLoS Biol. 2010 Jan 19;8(1):e1000291. doi: 10.1371/journal.pbio.1000291 (PMC2808216; doi:10.1371/journal.pbio.1000291)

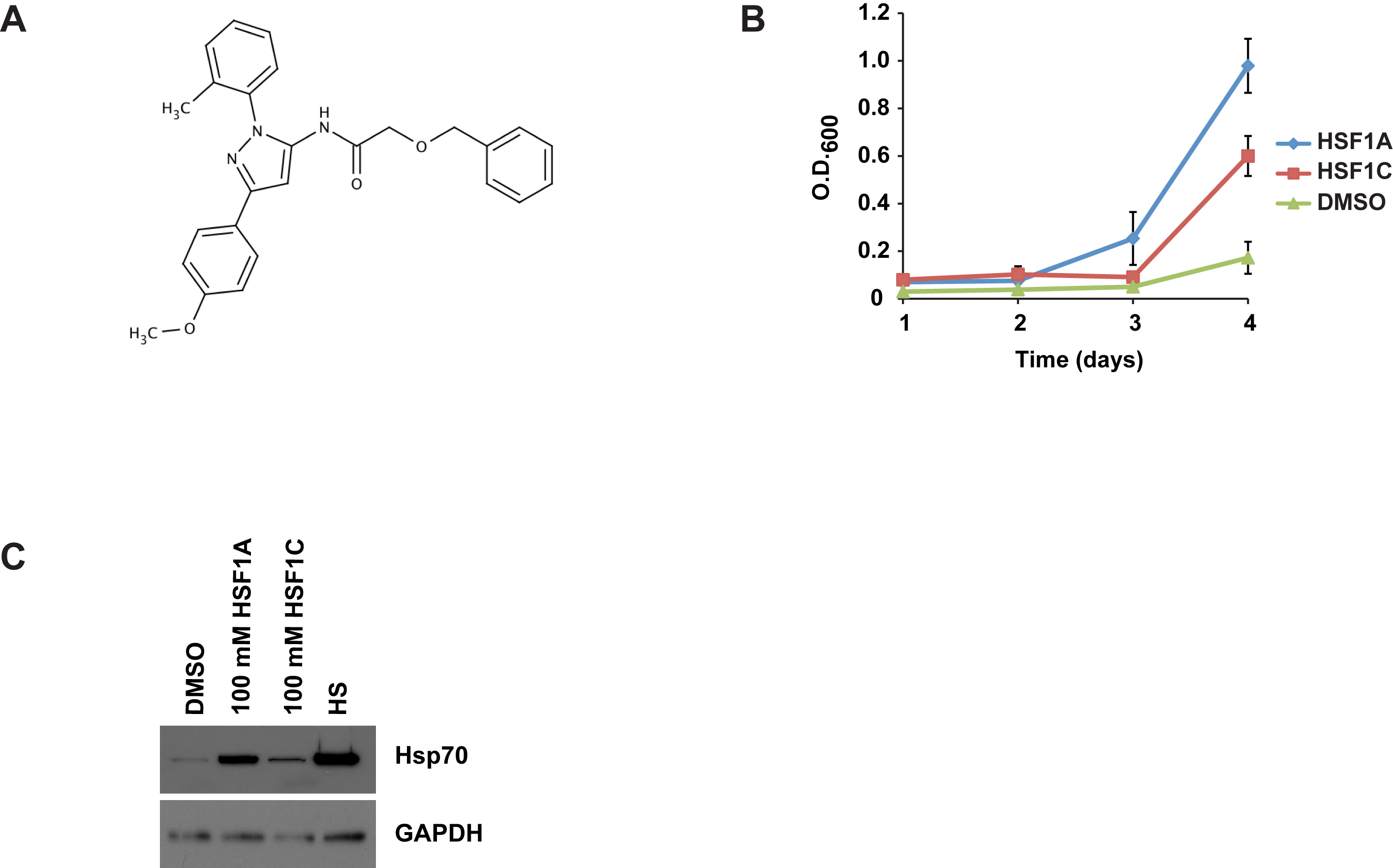

Supplement: Figure S1 — HSF1C activates human HSF1 function in yeast and mammalian cells. (A) Structure of HSF1C. (B) Yeast cells (DNY75) expressing wild-type human HSF1 were supplemented with 10 µM HSF1A, 10 µM HSF1C or DMSO, and grown in 96-well plates for 4 d. Growth was monitored by measuring OD600. (C) HSF1+/+ MEFs were treated with DMSO, 100 µM HSF1A, or 100 µM HSF1C for 15 h or heat shocked for 2 h at 42°C followed by a 15-h recovery. Total protein was analyzed for Hsp70 by immunoblotting. GAPDH serves as a loading control. (0.59 MB TIF) [file pbio.1000291.s001.tif]

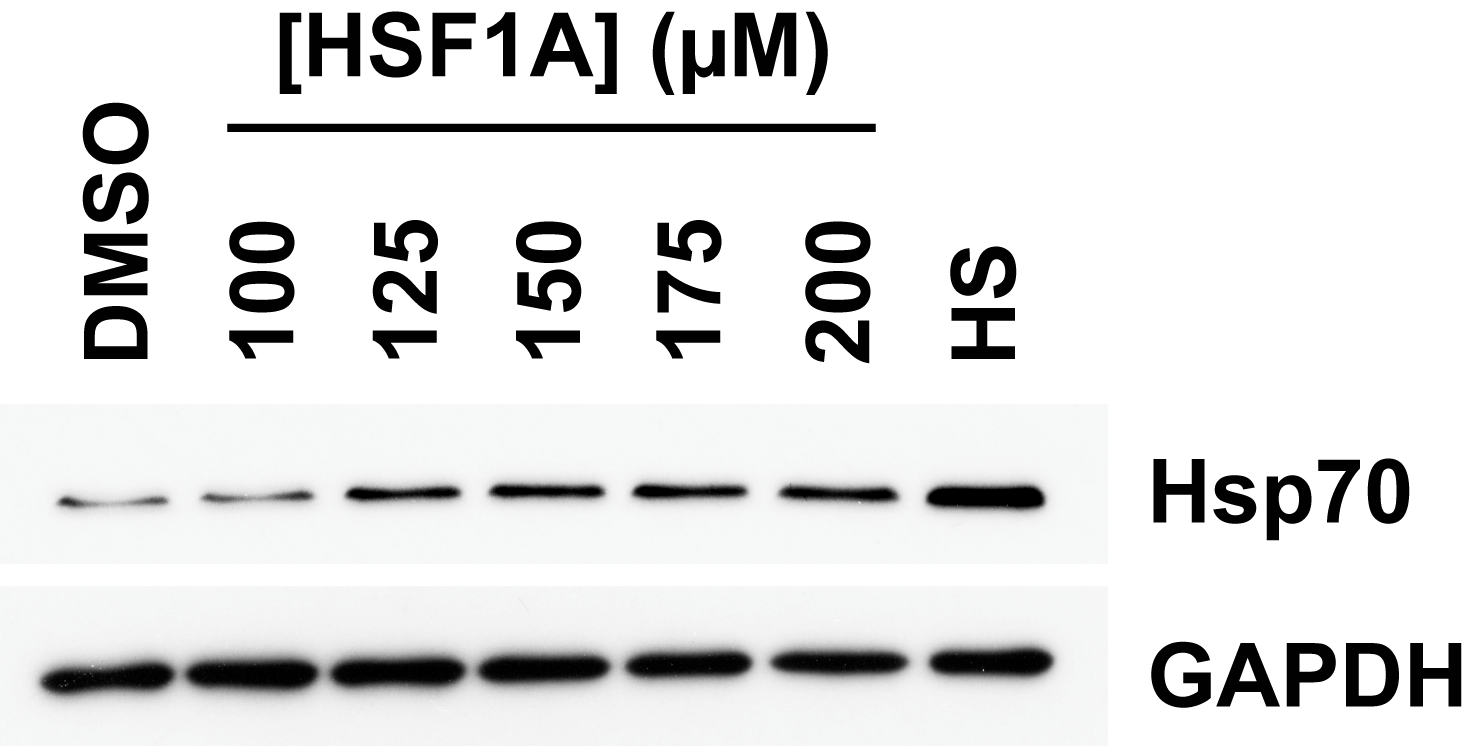

Supplement: Figure S2 — HSF1A promotes expression of Hsp70 in human cells. HeLa cells were treated with increasing concentrations of HSF1A for 15 h or heat shocked for 2 h at 42°C followed by a 15-h recovery. Total protein was extracted and analyzed for Hsp70 expression by immunoblotting. GAPDH serves as a loading control. (0.46 MB TIF) [file pbio.1000291.s002.tif]

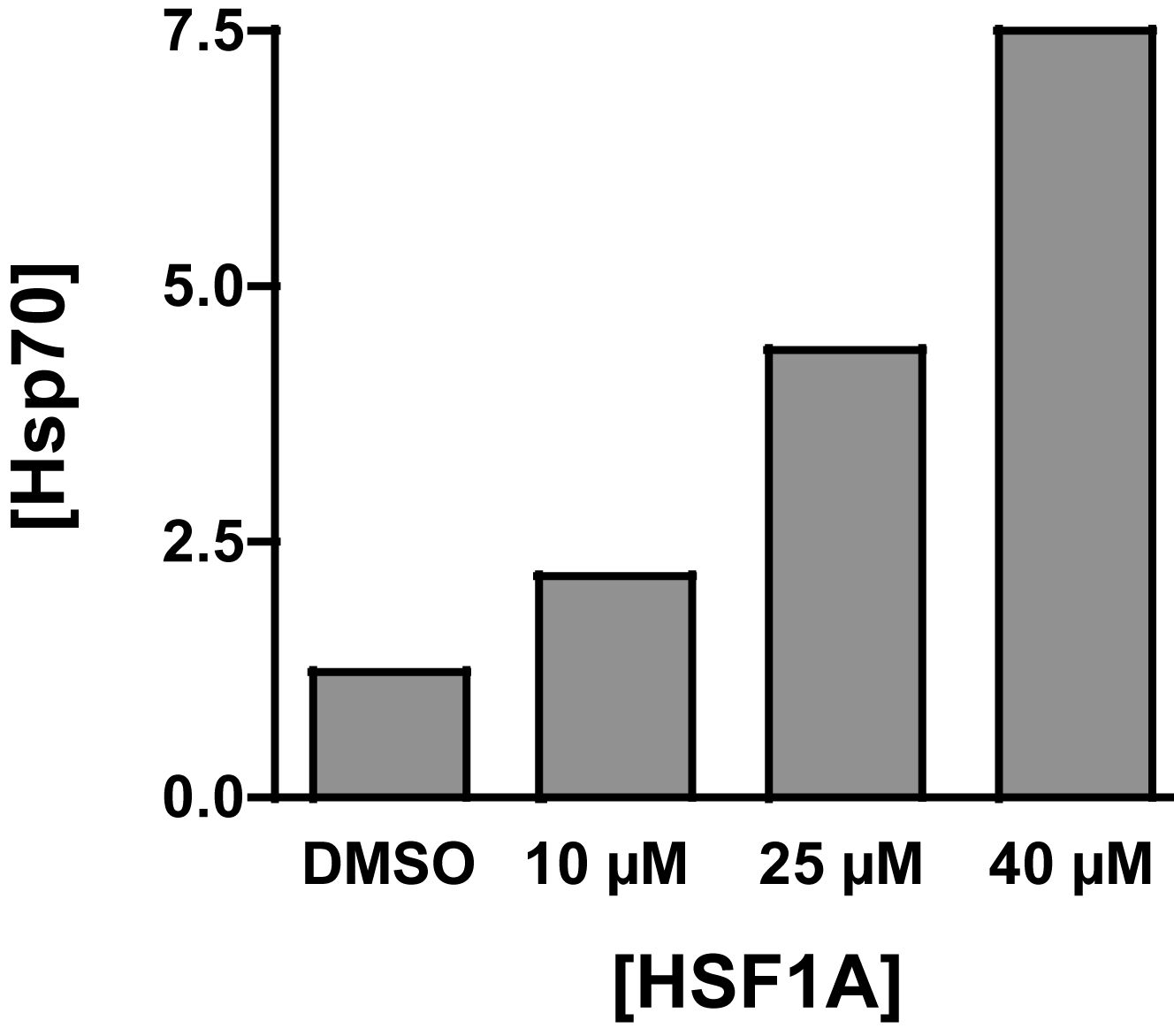

Supplement: Figure S3 — HSF1A promotes Hsp70 expression at low micromolar concentrations. PC12 cells were incubated with increasing concentrations of HSF1A for 72 h, and Hsp70 concentration was measured as a function of total protein concentration by ELISA (Assay Designs). (0.18 MB TIF) [file pbio.1000291.s003.tif]

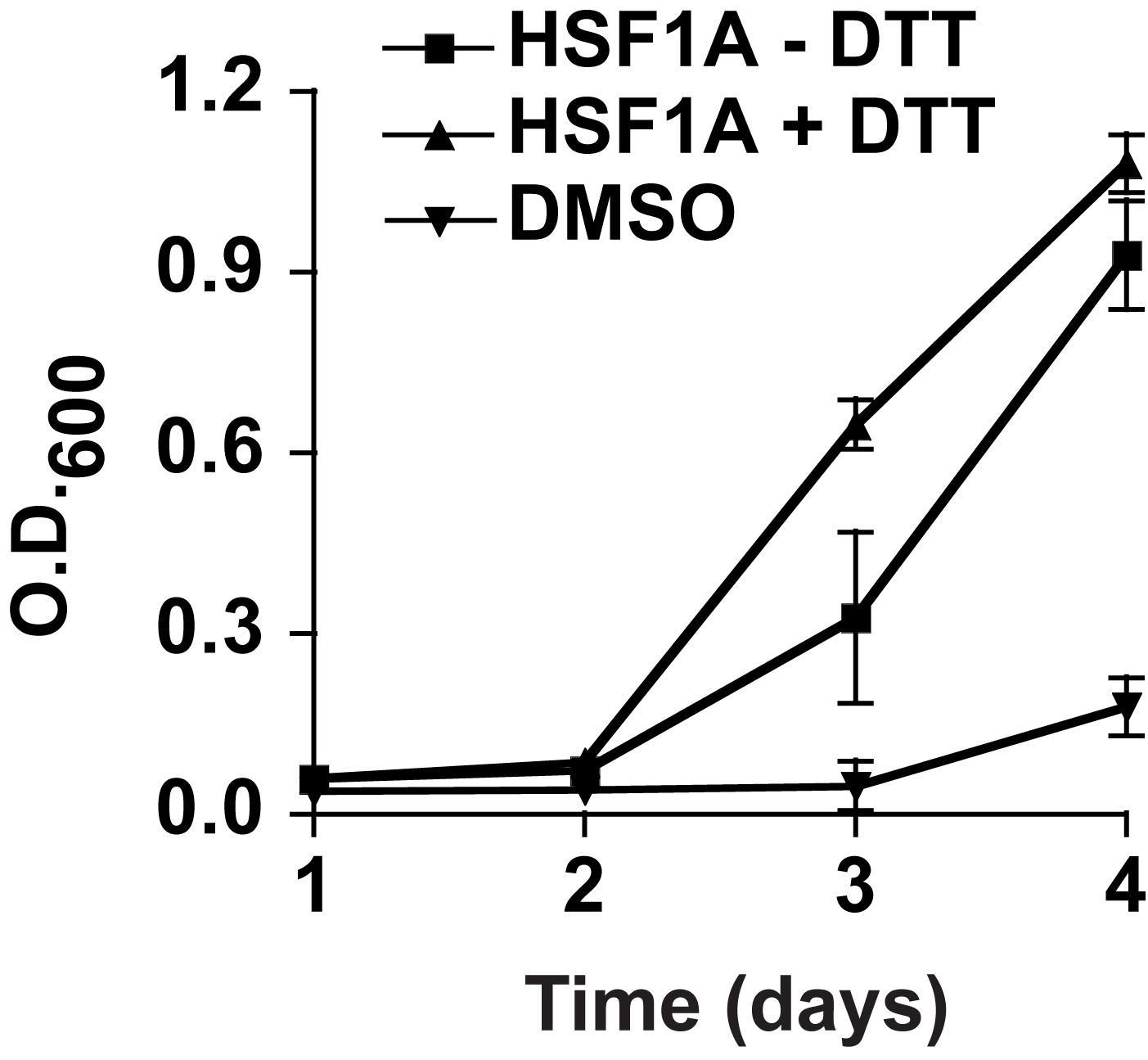

Supplement: Figure S4 — HSF1A-dependent activation of human HSF1 in yeast is not repressed by DTT. DNY75 cells were treated with 10 µM HSF1A in the absence or presence of 250 µM DTT and grown in 96-well plates for 4 d. Growth was monitored by measuring OD600. (0.26 MB TIF) [file pbio.1000291.s004.tif]

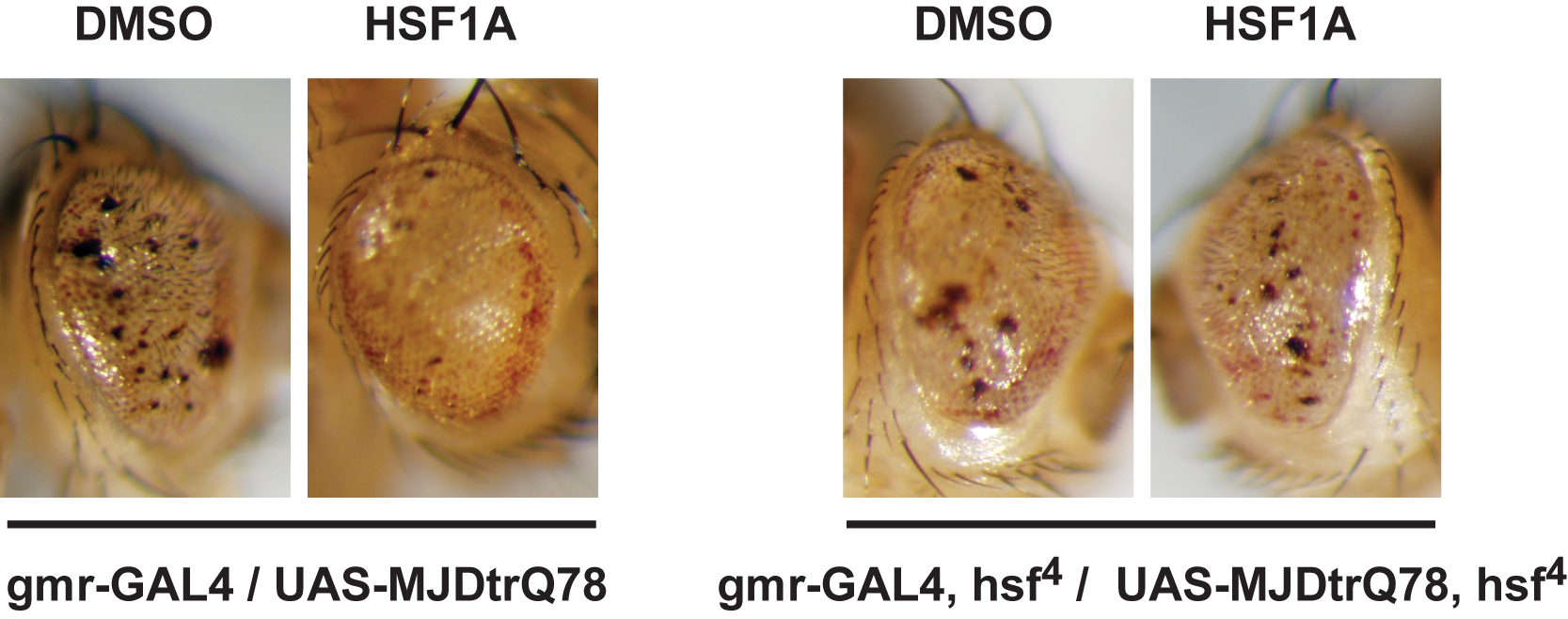

Supplement: Figure S5 — HSF1A does not reduce polyQ toxicity in flies carrying the hsf4 allele. hsf4, UAS-MJDtrQ78 recombinant flies were crossed to gmr-GAL4 flies in the chronic presence of food supplemented with DMSO or 400 µM HSF1A and maintained at 25°C, a semipermissive temperature for hsf4 activity. No reduction in polyQ-related phenotypes is observed in response to HSF1A treatment, suggesting that full HSF activity is required for HSF1A-dependent amelioration of polyQ induced phenotypes. (2.01 MB TIF) [file pbio.1000291.s005.tif]

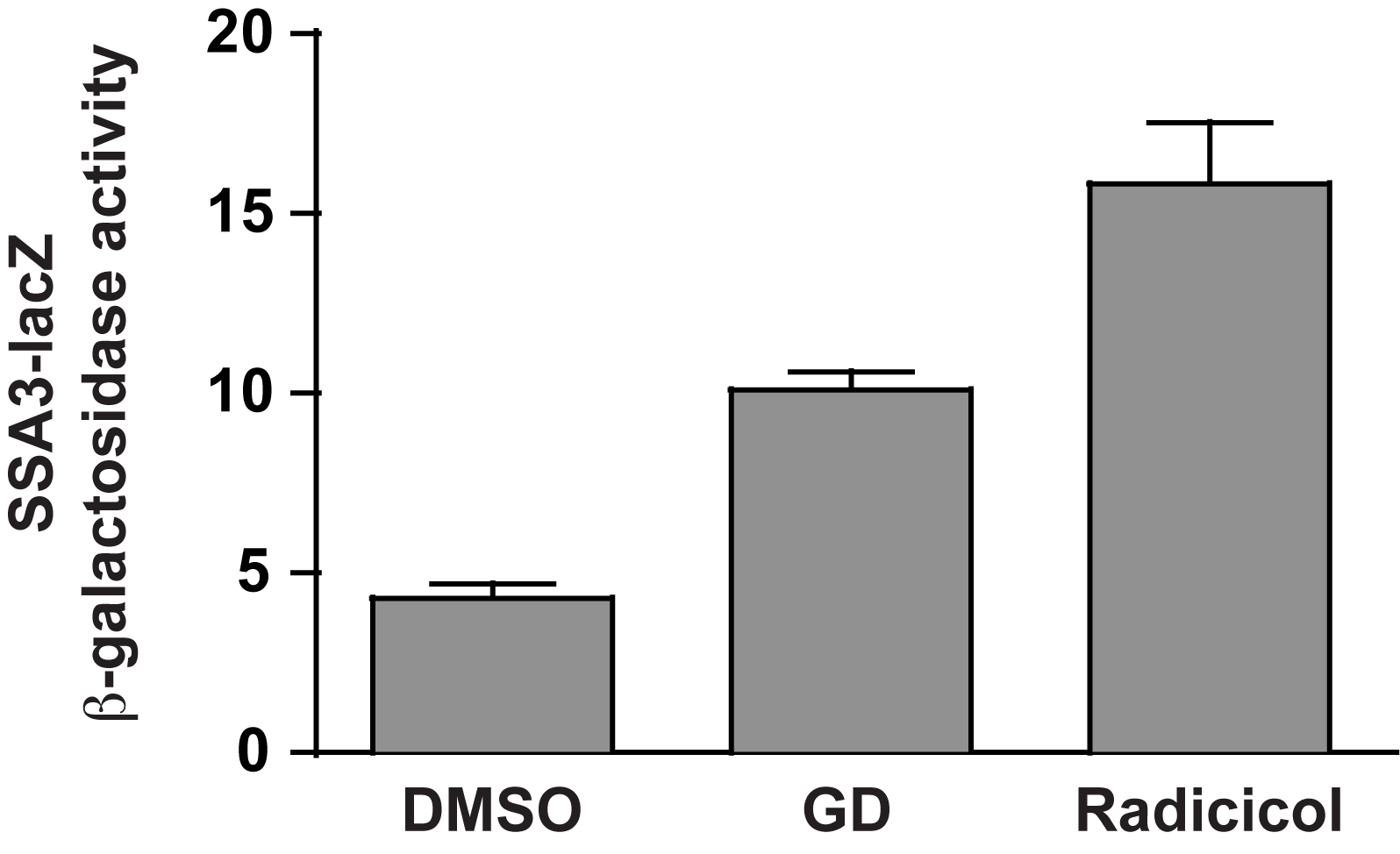

Supplement: Figure S6 — Geldanamycin and radicicol promote activation of SSA3-lacZ. Yeast strain DNY227, harboring the yHSF1-dependent SSA3-lacZ reporter gene, was exposed to DMSO, 10 µM geldanamycin (GD), or 10 µM radicicol for 3 h upon which time reporter gene activation was assessed by β-galactosidase activity assays. (0.26 MB TIF) [file pbio.1000291.s006.tif]

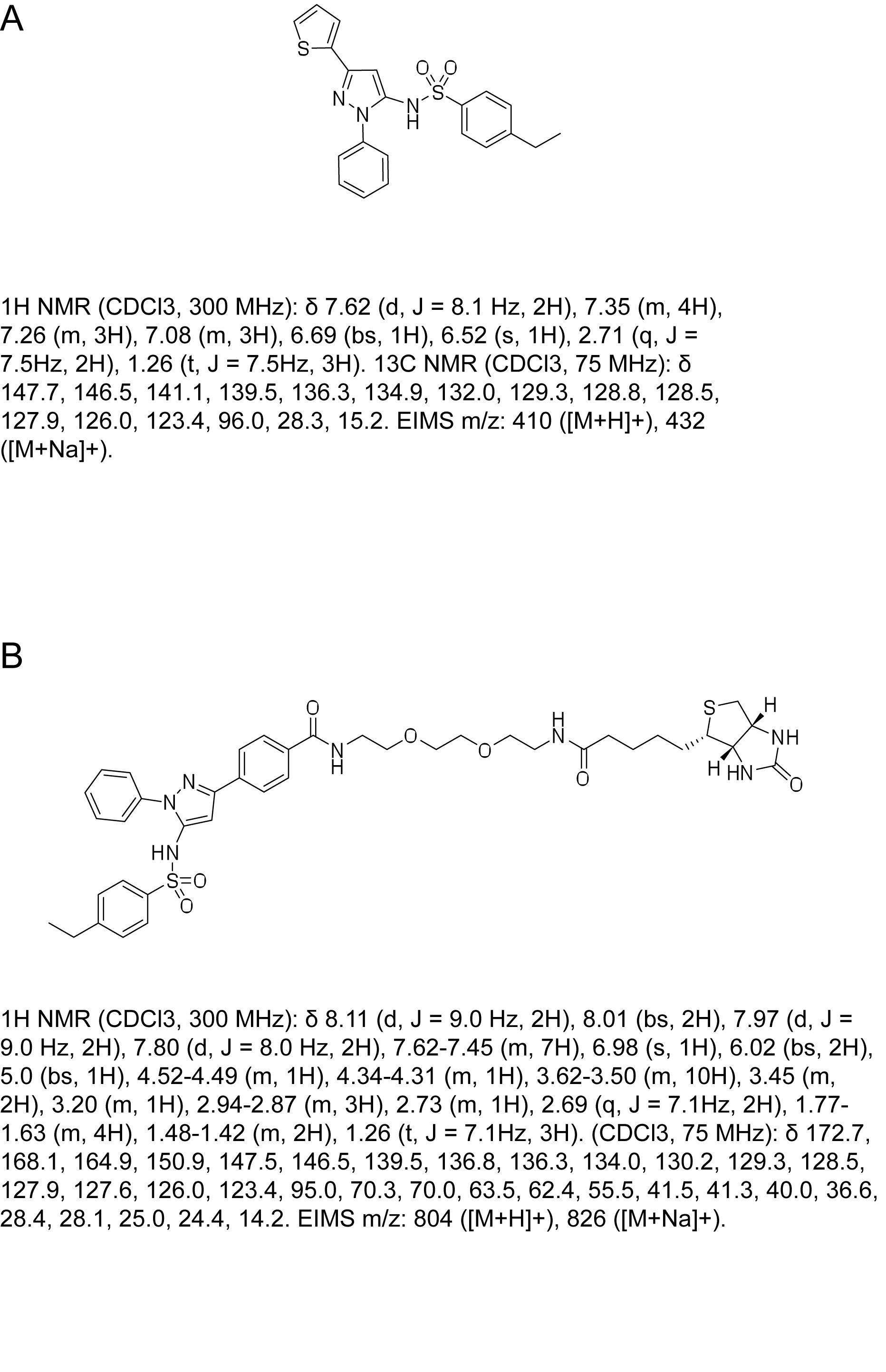

Supplement: Figure S7 — (A) Structure, 1H/13C, and EIMS data of HSF1A. (B) Structure, 1H/13C, and EIMS data of HSF1A-biotin. (0.62 MB TIF) [file pbio.1000291.s007.tif]

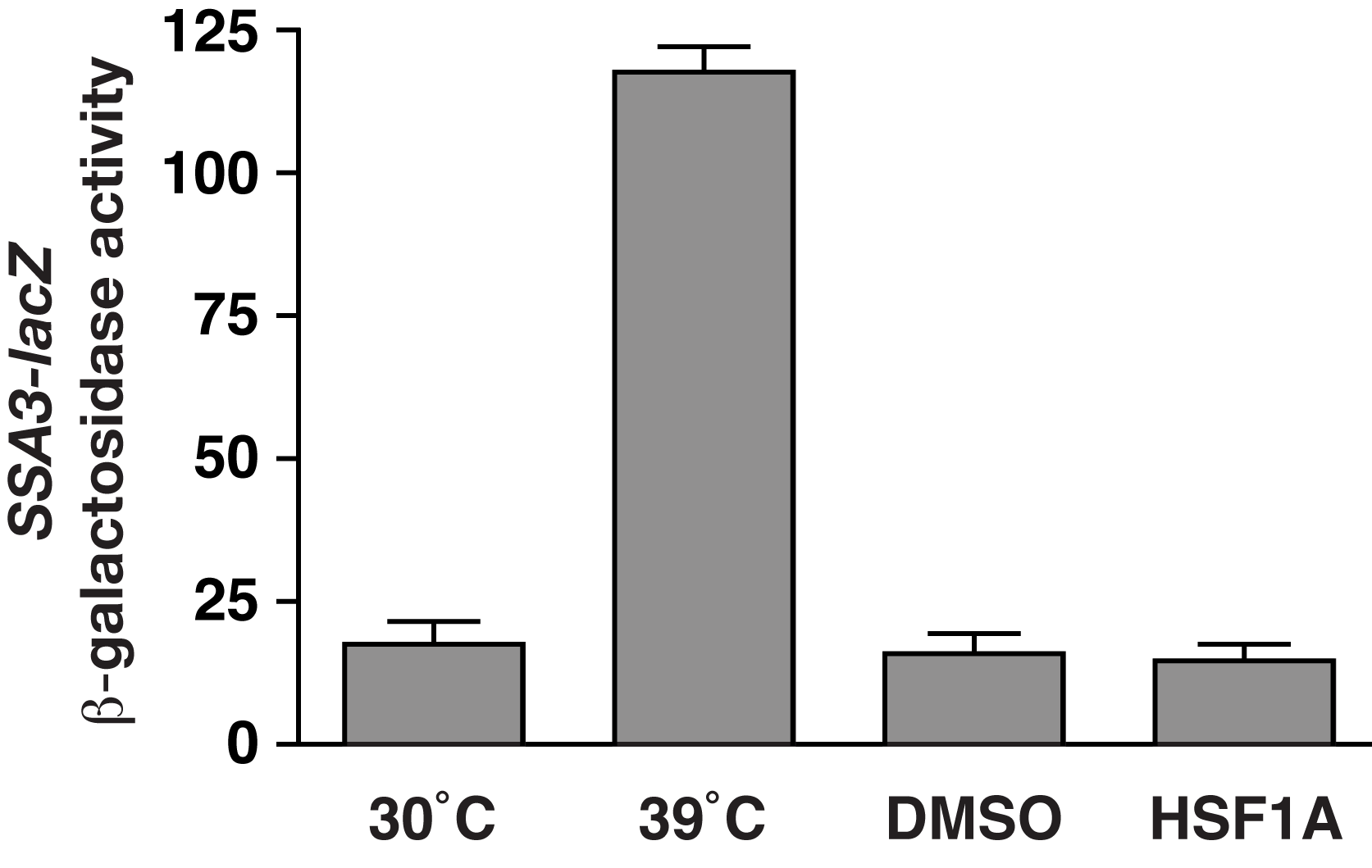

Supplement: Figure S8 — HSF1A does not activate yeast HSF. Yeast cells expressing the yHSF1-dependent SSA3-lacZ reporter gene were grown at 30°C and exposed to 20 µM HSF1A or DMSO for 6 h or heat shocked at 39°C for 3 h. Reporter gene activation was assessed by β-galactosidase activity assays. (0.29 MB TIF) [file pbio.1000291.s008.tif]

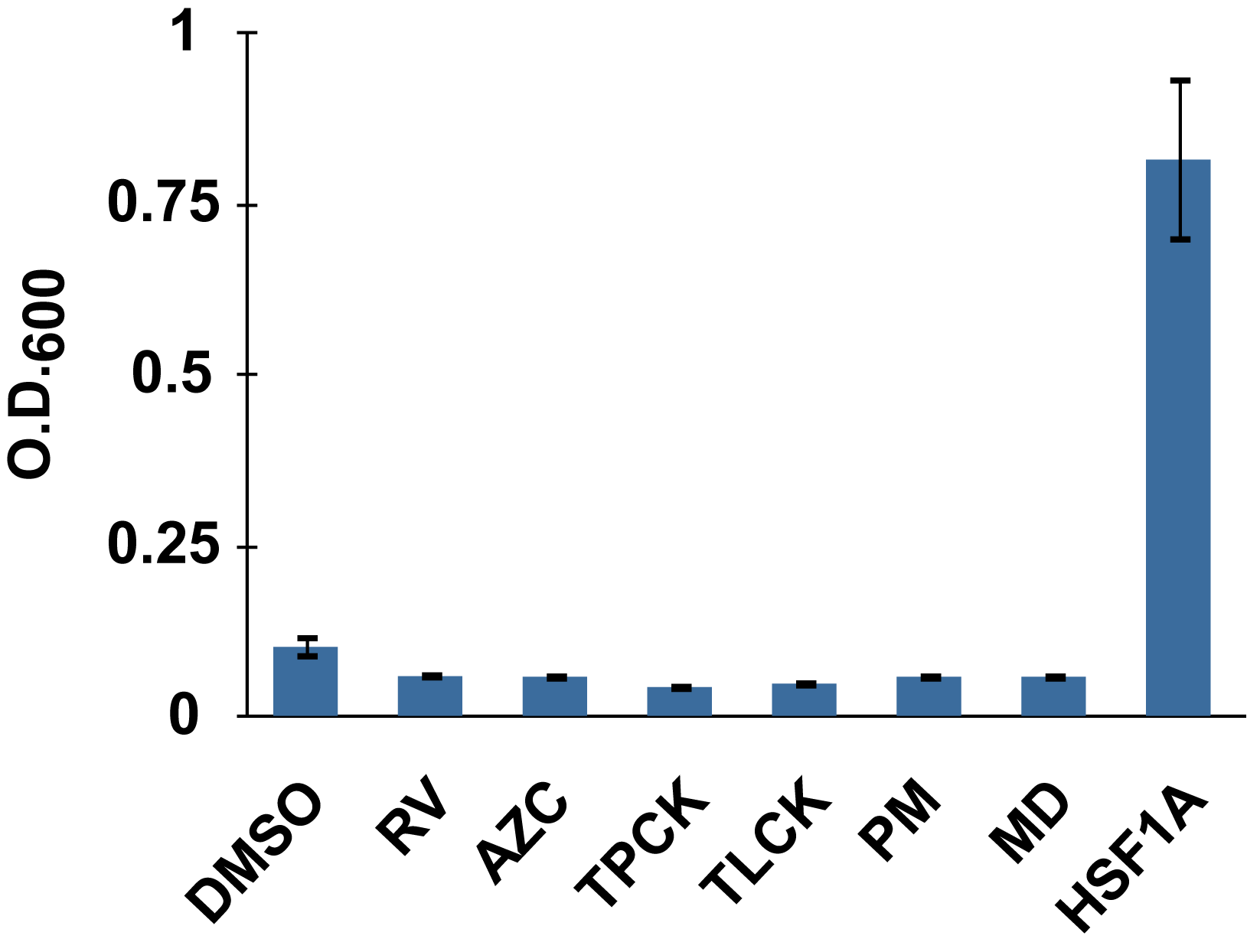

Supplement: Figure S9 — Human HSF1 is not activated in yeast by proteotoxic agents. Yeast cells were treated with either HSF1A, resveratrol (RV), azetidine (AZC), TPCK, TLCK, puromycin (PM), or menadione (MD) at a concentration of 10 µM and for 4 d. Growth was monitored by measuring OD600. OD600 readings at day 4 are shown. (0.25 MB TIF) [file pbio.1000291.s009.tif]
